# Analyzing fine scaling quantum effects on the buckling of axially-loaded carbon nanotubes based on the density functional theory and molecular mechanics method

**DOI:** 10.1038/s41598-024-55701-6

**Published:** 2024-03-28

**Authors:** M. Mirnezhad, R. Ansari, S. R. Falahatgar, P. Aghdasi

**Affiliations:** 1 Faculty of Mechanical Engineering, University Campus 2, University of Guilan, Rasht, Iran; 2 Faculty of Mechanical Engineering, University of Guilan, P.O. Box 3756, Rasht, Iran; 3 Department of Chemical and Materials Engineering, University of Alberta, Edmonton, AB T6G 2H5 Canada

**Keywords:** Fine scale, Quantum effects, Carbon nanotubes, Buckling strain, Quantum mechanics, Molecular mechanics, Graphene, Nanoscale materials, Quantum physics, Carbon nanotubes and fullerenes, Graphene, Structural properties, Two-dimensional materials

## Abstract

In this paper, the quantum effects of fine scaling on the buckling behavior of carbon nanotubes (CNTs) under axial loading are investigated. Molecular mechanics and quantum mechanics are respectively utilized to study the buckling behavior and to obtain the molecular mechanics coefficients of fine-scale nanotubes. The results of buckling behavior of CNTs with different chiralities with finite and infinite dimensions are given, and a comparison study is presented on them. The differences between finite and infinite nanotubes reflect the quantum effects of fine scaling on the buckling behavior. In addition, the results show that the dimensional changes highly affect the mechanical properties and the buckling behavior of CNTs to certain dimensions. Moreover, dimensional changes have a significant effect on the critical buckling strain. Beside, in addition to the structure dimensions, the arrangement of structural and boundary atoms have a major influence on the buckling behavior.

## Introduction

Since the pioneering discovery of carbon nanotubes (CNTs) by Iijima^1^, these nanomaterials have garnered significant attention owing to their exceptional characteristics encompassing stiffness, thermal conductivity, mechanical strength, and electrical properties, surpassing those of conventional materials^2–7^. However, due to the practical difficulties and high costs associated with experimental manipulation, the theoretical analysis and modeling of nanostructured materials have been a notable area of interest for many researchers^8^. To address the modeling challenges posed by nanostructured materials, scientists have employed diverse methodologies, including molecular dynamics (MD) simulations^9,10^, molecular mechanics (MM) models^11–14^ and continuum mechanics^15–17^. MD simulations, while effective, are computationally intensive and constrained by limitations in processing speed, making them suitable for small-scale problems with restricted time and length scales. The investigation of critical aspects such as nanotube wall thickness and bending stiffness remains a complex issue among researchers, prompting the establishment of a quasi-continuum framework to probe the nanomechanics of CNTs and tackle these challenges^18–20^. Molecular mechanics methods have proven especially valuable in elucidating the mechanical properties of carbon nanotubes^11,21–23^. These techniques involve the division of the overall energy into individual components, encompassing factors such as bond elongation, angle fluctuations, torsional effects, inversion, van der Waals forces, and electrostatic interactions^11^. The molecular structural mechanics approach represents a widely used strategy for investigating the characteristics of nanomaterials. This approach facilitates efficient computations with a high degree of precision, effectively bridging the gap between molecular mechanics and structural mechanics^24–26^. Since CNTs have long and hollow tubular structures, they are subject to buckling or structural instability^27^ which can affect their performance as structural or functional elements in nanoelectromechanical systems^28,29^ and CNT-based nanocomposites^29–32^. Thus, many researches have been conducted for the buckling analysis of CNTs under various loading conditions such as bending^33–40^, combined loading^41–45^, torsion^46–50^ and compression^51–57^.

Using MD simulations and continuum mechanics models, Yakobson et al.^9^ studied the buckling of SWCNTs under axial compression. It was seen that the cylindrical shell configuration of a SWCNT with an aspect ratio of 6 would not change but axial compression makes it shorter. The effects of the axial compression and bending on the critical buckling strain of multi-walled carbon nanotubes (MWCNTs) has been studied by Chang et al.^58^ using the molecular mechanics model. It was shown that only the part of the outer layers buckles first while the remaining inner part remains stable in a very thick MWCNT. In addition, investigation of the effects of tube size on the initial buckling wavelength revealed that the dependence of the initial buckling wavelength on the thickness of the MWCNT is low. Employing systematic MD simulations, Chang et al.^59^ studied the bending of the thin (three-walled) and thick (seven-walled) MWCNTs at low temperature of 1 K. It was shown that the initial buckling mode of thick and thin MWCNTs are different from each other and only several outer layers buckle first, while the rest of the inner layers remain stable in a very thick MWCNT. Using comprehensive MD simulations, Jeong et al.^48,49^ characterized the torsional behaviors of different CNT systems, such as bundled, chemically functionalized and nanotubes filled with other materials. Their results of Ref.^48^ demonstrated that the amount which affects the increase of the critical buckling moment relies on the number of inner tubes and the type of filling materials. The effect of initial stress on the torsional buckling behaviors of CNT systems was later examined by the authors^49^. They indicated that the critical torsional moment and stiffness could be significantly enhanced by the presence of initial stress. Using the nonlocal elasticity equations of Eringen, Pradhan and Reddy^60^ investigated the buckling of SWCNT with Winkler foundation. It was shown that as the size of SWCNT decreases, the nonlocal effects increase and as nonlocal parameter increases, load ratio’s decreases. In addition, further load ratio’s increases with increase in Winkler modulus (K) for clamped–clamped, simply supported, clamped hinged boundary conditions. However, load ratio’s decreases but with increase in Winkler modulus (K) for clamped free boundary condition. The buckling behavior of simply supported-simply supported single-walled carbon nanotubes (SWCNTs) was studied by employing the finite element method (FEM) by Bocko et al.^61^ for the nanotubes with and without defects. The carbon nanotubes were modeled as beams and shells and it was seen that the critical buckling force of SWCNTs would decrease as the number of defects increases. It was also seen that the SWCNTs with the same diameter but different chirality have almost the same decrease in the critical buckling force. Using continuum mechanics models and molecular mechanics simulation, Ma et al.^62^ studied buckling behaviours of the pre-stressed multi-walled carbon nanotubes (PS-MWCNTs) with two to six layers. They revealed three features of the buckling behaviour of PS-MWCNTs while the interlayer distance is considered as the key factor and the nanotubes are affected by the axial loading. It was demonstrated first, depending on the diameter of nanotubes, the buckling membrane force is not a monotonic function of interlayer distance. Second, the interlayer distance decreases for PS-MWCNTs with fixed intertube chirality as the buckling membrane force increases and third, the buckling membrane force increases as the number of walls increases for PS-MWCNTs with the same innermost tube. Moreover, they stated that the multi-shell continuum model and molecular mechanics simulation agree on the trend of the buckling membrane force as a function of interlayer distance, tube chirality index, and number of layers. Using molecular mechanics simulation surface Young’s modulus of SWCNTs with different chiral angles and diameters were calculated by Fang et al.^13^. And based on same method Wan et al.^63^ studied the mechanical properties of carbon nanotubes by developing a structural mechanics model. Their results stated that with the help of finite element analysis, the method they used is faster than atomistic simulations which makes it possible to simulate problems involving a large number of atoms. Using density functional theory calculations Chaudhuri et al.^64^ investigated a series of zigzag and armchair nanotubes of carbon, boron and nitrogen with various values of tube diameters to understand the effect of the diameter values and the chirality on the energetics, structure and electronic properties of nanotubes. It was revealed that carbon-boron (CBNT) and carbon–nitrogen (CNNT) nanotubes mainly shows metallic behavior based on the composition. While boron-nitrogen (BNNT) nanotubes seemed to have semiconducting behavior. In addition, the stability of the nanotubes were proven to be dependent on the respective chiralities. Hwang et al.^65^ investigated the mechanical behavior of multi-walled carbon nanotubes (MWNTs) utilizing the molecular dynamics (MD), under uniaxial tensile loading while MWNTs are fixed at both ends. The range of Young’s modulus of these nanotubes was found to be in the range between 0.85 and 1.16 TPa. Different radii and lengths of the tubes were stated as a possible reason to the difference in the obtained modulus. It was also found that changing the number of boundary layers would have no effect on the calculated mechanical properties, which indicates indicating the role of mechanical boundary conditions in the MD simulations. Cao^66^ developed carbon fiber-reinforced thermoplastic sandwich composites and introduced an innovative technique to enhance the interphase between the skin and core. This method involved the incorporation of resin-coated CNT-yarn fillers, offering a viable solution to improve the debonding toughness in low-weight-gain sandwich composites. The examination of fracture characteristics in reinforced concrete incorporating carbon nanotubes (CNTs) was conducted through the utilization of double-K fracture parameters by Zheng et al.^67^. The findings indicated that the incorporation of CNTs can enhance the material's resistance to cracking. Kim et al.^68^ systematically investigated alterations in the fiber microstructures, with a particular focus on the inter-bundle and intra-bundle voids during the process of solution spinning. Near the spinneret exit, it becomes evident that extensional deformation, achieved through drawing, plays a crucial role in orienting the fibers. Monavari et al.^69^ investigated the electronic response of single-walled carbon nanotubes (SWCNTs) and a carbon nanobelt (CNB) to N-linked and O-linked SARS-CoV-2 spike glycoproteins, using ab initio quantum mechanical approach. The CNTs were selected from three zigzag, armchair, and chiral groups. They examine the effect of carbon nanotube (CNT) chirality on the interaction between CNTs and glycoproteins. Results indicated that the chiral semiconductor CNTs clearly response to the presence of the glycoproteins by changing the electronic band gaps and electron density of states (DOS). Since the changes in the CNTs band gaps in the presence of N-linked were about two times larger than the changes in the presence of the O-linked glycoprotein, chiral CNT may distinguish different types of the glycoproteins. The same results were obtained from CNBs. Ma et al.^70^ showed that quantum defects do not affect aqueous two-phase extraction (ATPE) of different SWCNT chiralities into different phases, which suggests low numbers of defects. Interestingly, they observed a stochastic (Poisson) distribution of quantum defects. SWCNTs have most likely one to three defects (for low to high (bulk) quantum defect densities). These results show that there can be a large discrepancy between ensemble and single particle experiments/properties of nanomaterials. Mohammad et al.^71^ doped net CNTs with nonmetallic fluorine via a facile synthesis method to increase the efficiency of carbon perovskite solar cells (CPSCs). It was found that introducing fluorine-doped CNTs (F-CNTs) as hole-selective materials (HSMs) for MAPbI3 perovskite could reach up to an efficiency of 15.29%, higher than the efficiency of 13.70% in devices with a net CNT layer. By doping CNTs with fluorine, the charge-transfer resistance and series resistance were reduced, resulting in lower charge recombination at the perovskite/CNT interface Su et al.^72^ systematically investigated the effect of the chiral structures of SWCNTs on their electrical transport properties by measuring the performance of thin-film transistors constructed by eleven distinct (n, m) single-chirality SWCNT films. The results show that, even for SWCNTs with the same diameters but different chiral angles, the difference in the on-state current or carrier mobility could reach an order of magnitude. Further analysis indicated that the electrical transport properties of SWCNTs have strong type and family dependence. With increasing chiral angle for the same-family SWCNTs, Type I SWCNTs exhibited increasing on-state current and mobility, while Type II SWCNTs showed the reverse trend. The differences in the electrical properties of the same-family SWCNTs with different chiralities could be attributed to their different electronic band structures, which determined the contact barrier between electrodes and SWCNTs, intrinsic resistance and intertube contact resistance.

The term "fine scale" pertains to the diminutive proportions of a structure, where each individual component commences its expansion to give rise to the broader dimensions of the structure. At a fine scale, distinct characteristics and mechanical responses are evident in comparison to the expanded state. As the size and dimensions increase, these variations diminish, and the progression of these alterations depends on the specific material and its size and dimensions. With the elongation of the structure's length and dimensions, the properties and mechanical behavior of the material tend to converge towards a stable state. Given the extensive utility of nano-sized structures, especially in nanoscale applications, it is crucial to utilize the material's properties and mechanical behavior specific to the structure's designated size to enhance calculation accuracy and minimize errors. The quantum effects associated with finite scaling in very small dimensions assume significant importance. In certain instances, altering the structure's size can lead to substantial disparities in the properties and mechanical behavior, setting one material apart from another. To put it differently, in certain cases, rather than resorting to materials with varying resistance levels, the same material can be employed, but with different dimensional sizes, achieving the desired outcome. Since the effect of fine scaling on the buckling strain has never been investigated before, in this paper, the buckling strain of fine-scale and infinite single-walled carbon nanotubes with different chiralities under axial strain is obtained using the molecular mechanics method. In order to utilize molecular mechanics, their coefficients need to be obtained. In this paper, the mentioned coefficients for fine-scale structures are calculated by equating the energy obtained from quantum mechanics and molecular mechanics. Moreover, the critical buckling strains of finite and infinite nanotubes with different chiralities are obtained using the molecular mechanics method.The results of this study confirm that the size of the structure affects the buckling strength of the material and for very small structures, the characteristics related to the size of the structure should be used.

## DFT simulation details

The current study is based on the work of Ansari et al.^8^, which explained how the final form of equations used in this paper are computed. Thus, here we just present the necessary equations to avoid duplications. The mechanical properties of the graphene sheet based on the strain energy calculation is Acquired through Quantum-Espresso coding procedure^73^. The calculations are based on the density functional theory^74–89^ and generalized-gradient approximation (GGA) function is applied along with the Perdew-Burke-Ernzerhof (PBE) exchange correlation^90,91^ and the projector-augmented wave (PAW) potential is applied were employed for the self-consistent total energy calculations and geometry optimizations. After optimizing the initial parameters of structures’ input, the kinetic energy cutoff for the plane wave basis set has been converged to $80 Ry$. In order to compute kinetic energy cutoff, total energy variations versus cutoff energy variations have been computed and we plot the variations. Finally, in cutoff energy $80 Ry$, difference in total energy versus cutoff energy reached lower than 0.005 eV. Hence, we selected $80 Ry$ as our optimized cutoff energy. The same trend we employed on optimizing k-point grid. The Brillion Zone was sampled using a $20\times 20\times 1$ Monkhorst Pack k-point grid. The basic concepts and relations used in the Quantum-Espresso code are expressed in the literature^8^.

## Molecular mechanics model

### Potential energy

**Figure 1:**
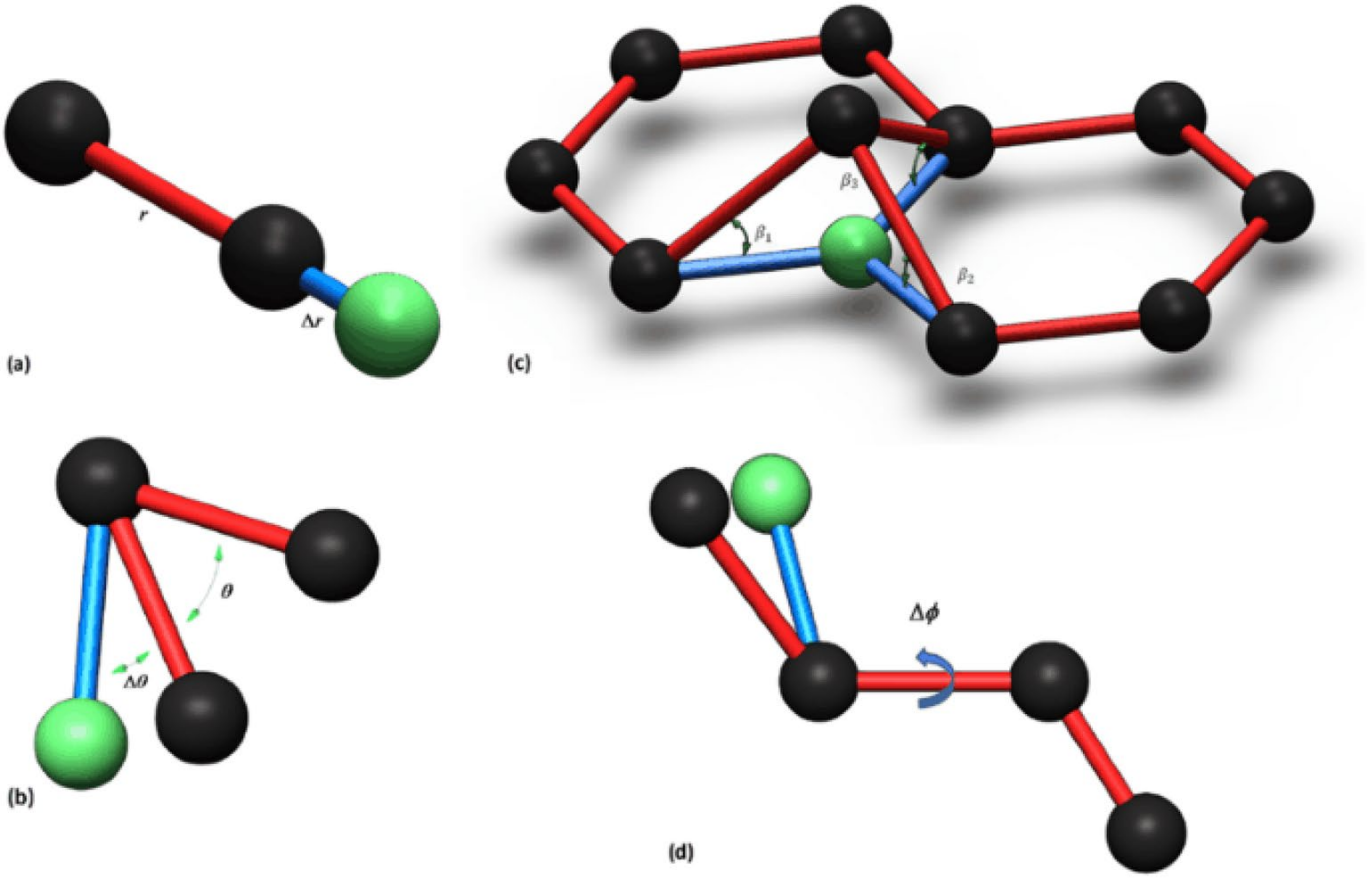
Different bonds structure of a C–C cell corresponding to energy terms (**a**) ${U}_{\rho }$, (**b**) ${U}_{\theta }$, (**c**) ${U}_{\omega }$, (**d**) ${U}_{\tau }$.

The total potential energy,${{\text{E}}}_{{\text{t}}}$, in the empirical force field method of molecular mechanics, can be stated as the sum of several energies due to valence of bonded interactions or bonded and non-bonded interactions (Fig. 1):

1 $${E}_{t}={U}_{\rho }+{U}_{\theta }+{U}_{\omega }+{U}_{\tau }+{U}_{vdw}+{U}_{es}.$$

In which ${U}_{\rho }$*, *${U}_{\theta }$*,* ${U}_{\omega }$, and ${U}_{\tau }$ are energies associated with bond stretching, bond angle variation, bond inversion, and torsion, respectively; ${U}_{vdw}$ and ${U}_{es}$ are also associated with van der Waals and electrostatic interactions, respectively^11,92–95^. Depending on the particular materials and loading conditions considered, different functional forms might be utilized for these energy terms. For the present buckling problem of carbon nanotubes, the terms ${U}_{\rho }$, ${U}_{\theta }$ and ${U}_{\omega }$ are expected to be significant in the total potential energy of the system. Furthermore ${U}_{vdw}$ can be neglected because SWCNTs are considered herein. As Hooke’s law has proven to be efficient and precise enough to describe the behavior of atoms under small deformation^94^, it is frequently employed to characterize the interactions between bound atoms in the system, therefore, using Ref.^96^, Eq. (1) may be written in the form:

2 $${E}_{t}=\frac{1}{2}\sum_{ij}\frac{1}{2}{K}_{\rho }\sum_{k}{({dr}_{ijk})}^{2}+\sum_{ij}\frac{1}{2}{C}_{\theta }\sum_{k}{({d\theta }_{ijk})}^{2}+\sum_{ij}\frac{1}{2}{C}_{\omega }{(\frac{1}{3}\sum_{k}{\beta }_{ijk})}^{2}.$$

Here the subscript $k =1, 2, 3$. Besides, it should be remarked that the constant coefficient $\frac{1}{2}$ of the first term in Eq. (2) is to ensure that bond stretching energy is considered only once.

### Axisymmetric buckling of single-walled chiral nanotubes

The structure of a single-walled carbon nanotube is often characterized by a pair of integers )$n,m$ (, representing its helicity^97^. The geometrical parameters are same as Sect. “Molecular mechanics model” of Ref.^96^. A carbon nanotube under compression tends to buckle when its axial strain exceeds the critical compressive strain,${\varepsilon }_{0}$ .Prior to buckling, the variation of bond length, bond angle and inversion angle from equilibrium values are obtained as Eqs. (9) to (27) of Ref.^8^. Then, using Eq. (24) of the mentioned reference, one would have:

3 $${dr}_{ij1}=\frac{{{\varepsilon }_{f}}^{p}{r}_{1}}{\frac{{\mathit{cot}\left(\frac{{\theta }_{3}}{2}\right)}^{2}{\lambda }_{A}{K}_{\rho }{r}_{1}^{2}}{{C}_{\theta }}+1}.$$

${dr}_{ij2}$ is also obtained as same as above approach:

4 $${dr}_{ij2}=\frac{{{\varepsilon }_{f}}^{p}{r}_{2}}{\frac{{\mathit{cot}\left(\frac{{\theta }_{3}}{2}\right)}^{2}{\lambda }_{A}{K}_{\rho }{r}_{2}^{2}}{{C}_{\theta }}+1}.$$

${{\text{dr}}}_{{\text{ij}}3}$ is obtained By means of Eqs. (13)–(15), (16), (18), (21) of Ref.^8^:

5 $${dr}_{ij3}=\left(\frac{\mathit{tan}\left(\frac{\pi }{6}-\Theta \right)}{{K}_{\rho }}\right)\left({C}_{\theta }{d\theta }_{3}+{C}_{\theta }{d\theta }_{1}\frac{\mathit{tan}\left(\frac{{\theta }_{3}}{2}\right)}{\mathit{tan}\left({\theta }_{2}\right)}\right)\left(\frac{1}{\mathit{cos}\left(\frac{{\theta }_{3}}{2}\right)\left(\frac{{r}_{3}}{2}\right)}\right),$$

6 $${d\theta }_{ij1}=-\frac{{d\theta }_{ij3}\mathit{sin}\left(\frac{{\theta }_{3}}{2}\right)\mathit{cos}\left(\frac{\pi }{n+m}\right)}{2\mathit{sin}\left({\theta }_{2}\right)},$$

7 $${d\theta }_{ij2}=-\frac{{d\theta }_{ij3}\mathit{sin}\left(\frac{{\theta }_{3}}{2}\right)\mathit{cos}\left(\frac{\pi }{n+m}\right)}{2\mathit{sin}\left({\theta }_{2}\right)},$$

8 $${d\theta }_{ij3}=\frac{{dr}_{ij1}}{{r}_{1}}\frac{\mathit{cot}(\frac{{\theta }_{3}}{2})(2{\lambda }_{A}{K}_{\rho }{r}_{1}^{2})}{{C}_{\theta }}.$$

According to the displacement of the atoms of SWCNT after buckling, the variations of bond length (${\Delta r}_{ij1} ,{\Delta r}_{ij2} ,{\Delta r}_{ij3}$), bond angle (${\Delta \theta }_{ij1} ,{\Delta \theta }_{ij2} , {\Delta \theta }_{ij3}$), and inversion angle (${\Delta \beta }_{ij}$) of the deformed SWCNT after buckling can be obtained. These quantities are given in the Appendix A of Ref.^96^. in which the following parameters are used,

9a $${\alpha }_{1}=0,$$

9b $${\alpha }_{2}=\mathit{arcsin}\left(\frac{{r}_{3}}{2R}\mathit{sin}\left(\frac{\pi }{3}+\Theta \right)\right),$$

9c $${\alpha }_{3}=\mathit{arcsin}(\frac{{r}_{1}}{2R}\mathit{sin}(-\frac{{\theta }_{3}}{2}+\frac{\pi }{3}+\Theta )),$$

9d $${\alpha }_{4}=\mathit{arcsin}\left(\frac{{r}_{2}}{2R}\mathit{sin}\left(\frac{{\theta }_{3}}{2}+\frac{\pi }{3}+\Theta \right)\right),$$

where $R$, the tube radius can be expressed in the form,

10 $$R=\frac{{r}_{0}\sqrt{3({n}^{2}+nm+{m}^{2})}}{2\pi },$$

where ${\xi }_{i,j}$ the radial displacement of the atom $ij$, is assumed to be small in comparison with ${r}_{0}$ .For atoms located at type $B$ position as shown in Fig. 5 of the Ref.^96^, the term ${ \xi }_{i,j+1}$ in the preceding equations must be replaced by ${\xi }_{i,j-1}$. In fact, there is no crucial difference between atoms at type $A$ and type $B$ positions. For convenience, the mathematical procedure described herein is only for atoms at a type $A$ position.

### Stability equation

Making use of the parameters discussed in Sect. “DFT simulation details”, the potential energy of the system prior to buckling, $dE$, and after buckling, $\Delta E$, can be computed with using Eq. (2) The free energy of the system, $\Pi$, is,

11 $$\Pi =dE-\Delta E$$

The condition for $\Pi$ to be extremum requires,

12 $$\frac{\partial \Pi }{\partial {\xi }_{ij}}=\frac{\partial (dE-\Delta E)}{\partial {\xi }_{ij}}=0$$

### Buckling strain

In the case of axisymmetric buckling, the radial displacement of atom ${\text{ij}}$ is of the form

13 $${\xi }_{ij}=Z\mathit{cos}\left(\frac{{m}_{1}\pi {x}_{ij}}{L}\right)+{Z}_{0}.$$

In which $L$ is the tube length, ${x}_{ij}$ is the longitudinal coordinate of atom $ij$, and ${Z}_{0}$, the rigid radial extension of the nanotube under a compressive strain of ${\varepsilon }_{0}$^12^.

14 $${Z}_{0}=\frac{8R{\varepsilon }_{0}}{3[3+{\lambda }_{A}(\frac{{K}_{\rho }{r}_{0}^{2}}{{C}_{\theta }})]}.$$

Introducing Eq. (12) into (13) yields the stability equation from which the buckling strain can be attained for its nontrivial solutions, $Z\ne 0$.

### Force constants (${K}_{\rho }$, ${C}_{\theta }$ and ${C}_{\omega }$), Young’s modulus, Poisson’s ratio and flexural rigidity of fine-scale structures

**Figure 2:**
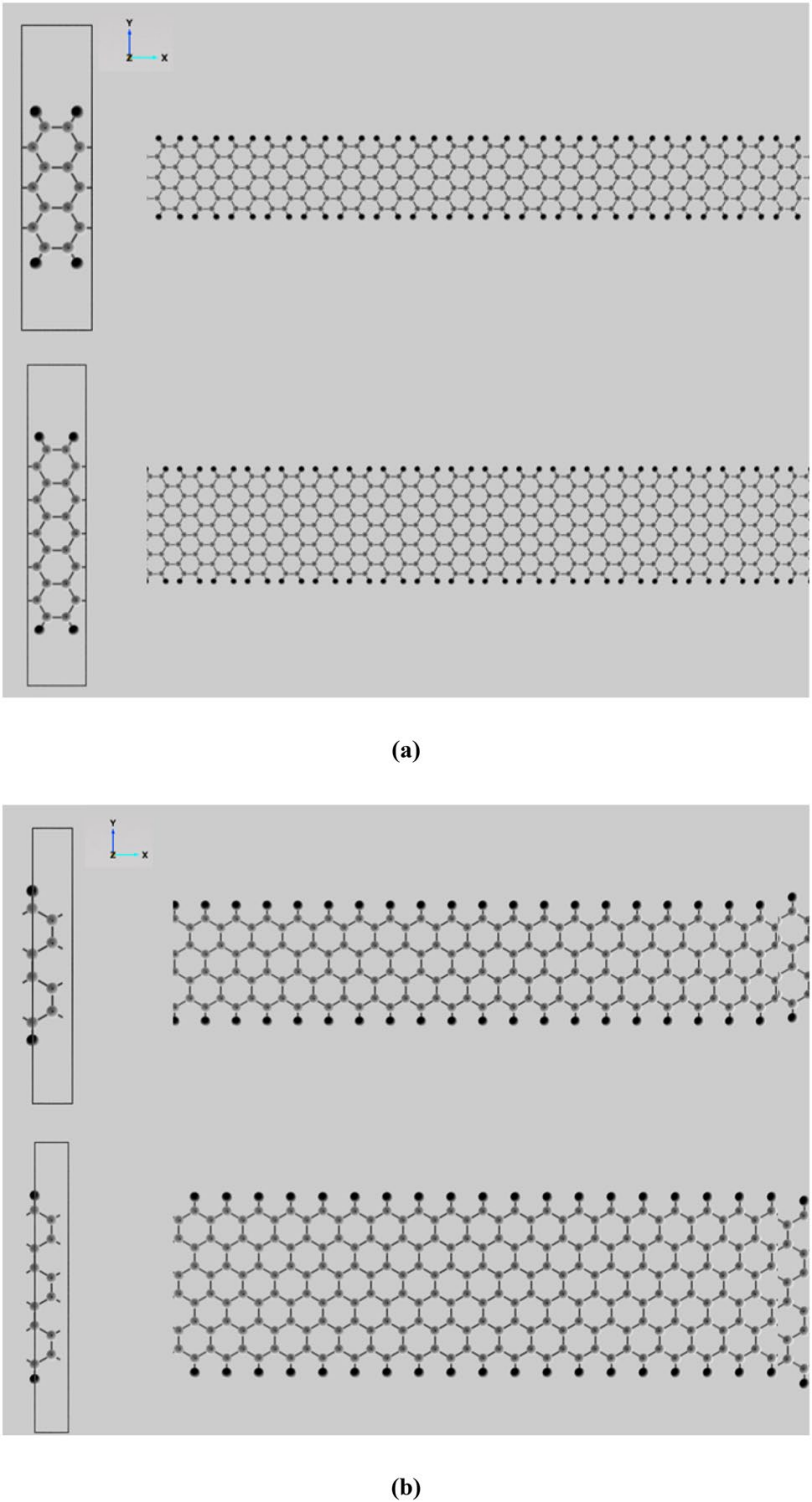
One-dimensional carbon nanostructures with specified width and (**a**) armchair and (**b**) zigzag boundary atoms.

To investigate the buckling behavior and other properties related to the structural energy of materials using molecular mechanics, the most important factor is to derive the coefficients of these equations in order to find the changes in energy values due to changes in material size and structural atomic arrangement. In this section, in order to account the fine-scale nature of the materials on its structural properties and to study buckling behavior and other energy related issues that can be extracted from molecular mechanics equations, the coefficients of appropriate molecular mechanics equations must first be extracted. To achieve this, due to the properties of fine-scale nanosheets, which leads to the creation of nanotubes with specified length, the molecular mechanics coefficients related to fine-scale structures have been extracted. The characterization of graphene as a two-dimensional carbon nanostructure that can be used as a standard for comparing the change of properties with respect to dimensional changes has also been studied. Structural energy variations were nonlinear and more varied widths should be selected in areas where changes are more frequent and in areas where energies are closer to 2D structures, points should be selected in accordance with this trend. For this purpose, we first need to select specific widths of the nanosheets along the desired directions, which can be used to characterize the properties change process as well as to optimize the calculations. As a result, in order to achieve this purpose, there are some widths considered in this paper whose shape are presented in Fig. 2 and their properties are presented Table 1.

**Table 1:** Effective width for investigation of mechanical properties characterizing of the fine-scale structures.

Nanoribbon structure with specified width	Length with excluding hydrogen atoms (Å)	Width with excluding hydrogen atoms (Å)	Area with excluding hydrogen atoms (Å^2^)	Number of carbon atoms	Density ($\mu g/{m}^{2})$
2a	7.383	4.262	31.470	18	1140.719
3a	12.304	4.262	52.451	26	988.625
5a	22.149	4.262	94.412	42	887.226
8a	36.914	4.262	157.353	66	836.529
2z	7.104	2.461	17.483	10	1140.719
3z	11.366	2.461	27.973	14	998.129
5z	19.891	2.461	48.954	22	896.282
9z	36.942	2.461	90.915	38	833.603

Widths 2a, 3a, 5a and 8a are selected in the armchair direction and 2z, 3z, 5z and 9z in the zigzag direction so that the widths are as close as possible to each other in terms of structure characteristic so that a closer comparison can be made between the effects of different directions. It should be noted that the points were chosen so that they would have higher abundance in less than 20 angstroms, where the energy variations were with respect to the size difference noticeable. Also around and higher than 20 angstroms, some points are selected to compare the properties and extent of changes. After determining the mechanical properties of the plate nanostructures according to the references^8,96^, the molecular mechanics coefficients are obtained, and the results of the mechanical properties and molecular mechanics coefficients are presented in Table 2.

**Table 2:** Molecular mechanics coefficients for graphene structure and fine-scale nanostructures.

Type of structure	Young’s modulus $(Gpa\times nm)$	Poisson’s ratio	${k}_{\rho }({\text{nN}}/{\text{nm}})$	${C}_{\theta }$ (${\text{nN}}\times {\text{nm}}$)	Cω (nNnm)
Graphene	350	0.016	721.687	1.376	1.376
2a	370.017	0.056	1456.567	0.803	0.610
3a	361.315	0.047	1180.785	0.872	0.698
5a	355.662	0.033	919.440	1.040	0.770
8a	351.034	0.030	868.585	1.075	0.814
2z	431.282	0.041	1266.107	1.125	0.594
3z	398.552	0.027	945.635	1.281	0.653
5z	371.917	0.023	836.596	1.280	0.713
9z	352.210	0.022	782.110	1.235	0.747

## Numerical results and discussion

Using the following formula buckling mode parameter $\xi$ can be obtained:

15 $$\xi =\frac{\sqrt{3}{m}_{1}\pi {r}_{0}}{2L}.$$

Here, $L,{r}_{0}$ and ${m}_{1}$ represent the tube length, $C-C$ bond length and the number of half waves in the axial direction respectively. It is clear that the minimum value of the buckling strain (i.e. the critical buckling strain) is sensitive to the tube diameter so that the critical buckling strain decreases with the increase of tube diameter. By introducing the buckling wavelength

16 $$l=\frac{\sqrt{3}\pi {r}_{0}}{\xi }.$$

It can be observed that the critical buckling strain occurs at higher buckling mode parameter (or at lower wavelength), as previously reported in Ref.^12^.

**Figure 3:**
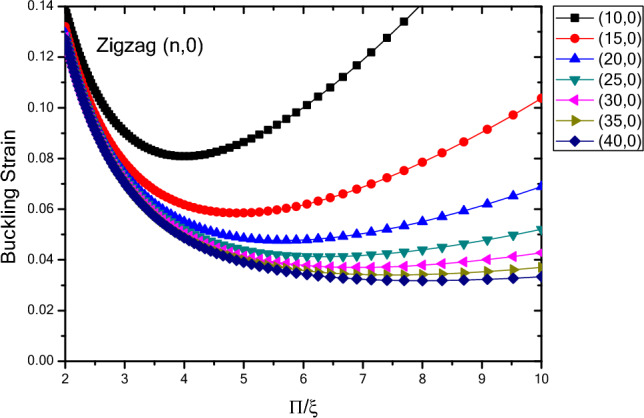
Buckling strain values $\left({\upvarepsilon }_{0}\right)$ of infinite length $\left(n,0\right)$ nanotubes with zigzag atomic arrangement with respect to inverse buckling mode parameter $\xi$.

In Fig. 3, the values of the buckling strain ${(\upvarepsilon }_{0})$ of infinite tubes with zigzag atomic arrangement and chirality of $\left(n,0\right)$ are shown in terms of inverse mode modulus $(\xi )$. The results show that by varying the buckling mode parameter from maximum value to minimum, the buckling strain values also change to a minimum value. This value can be considered as the critical buckling strain. The results also show that for the larger buckling mode parameters, the maximum buckling strain values are larger than the similar values for the smaller buckling mode parameters over the study period. In addition, for nanotubes with zigzag atomic arrangement, the buckling strain values change from a smaller and closer range to a wider range with decreasing buckling mode parameter value. It can be seen that as the diameter of the nanotube structure increases, the extent of buckling strain increases and the buckling strain values decrease. In smaller buckling mode parameters, the buckling strain is more dependent on the diameter and decreases with increasing the diameter. Moreover, it is seen that by increasing the diameter of nanotube, critical buckling strain occurs in smaller buckling mode parameter and the critical buckling strain decreases. In addition, as the diameter of the buckling strain changes, the buckling mode becomes closer to each other. Furthermore, it is seen that in the larger buckling mode parameters the variation of changes in the buckling strain are higher and lower in the smaller buckling mode parameters.

**Figure 4:**
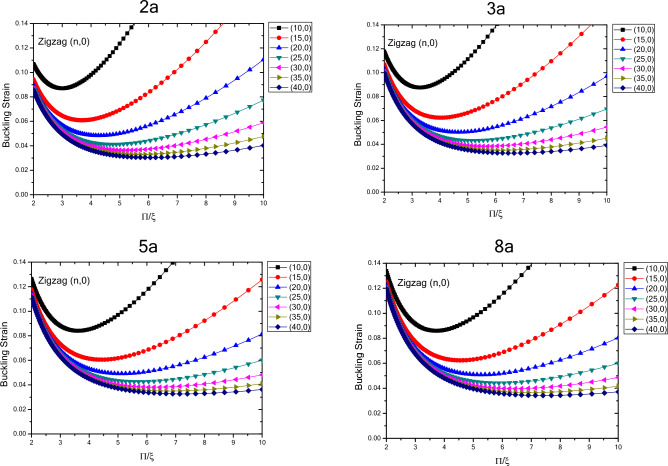
Changes in buckling strain values of zigzag nanotubes with specified lengths obtained by wrapping armchair nanosheets with respect to inverse buckling mode parameter.

The results of variations in buckling strain values of zigzag nanotubes with specified lengths obtained by wrapping armchair nanosheets are presented in terms of inverse buckling mode parameter are plotted in Fig. 4. By comparing the results, the buckling strain susceptibility of zigzag nanotubes from dimensional changes and quantum effects of fine scaling will be determined. The results show that by increasing the length of the structure in a well-defined process, the buckling strain values start from smaller values and increase as the length of the nanotubes increase. For smaller nanotubes, the buckling strain values for the larger buckling mode parameters have a wider range of values, which increase by increasing buckling mode parameter. It can be concluded that as the length of the structure increases, the range of the buckling strain values increases. In other words, by increasing the length of the structure and the diameter of the structure, the buckling strain is affected by the quantum effects of fine scaling, which is greater for the diameter variations compared to the longitudinal variations. The curvature effects can also be considered effective in this case. The results show that due to variations in the buckling mode parameter, the range of buckling strain values in nanotubes with larger lengths have increasing trend. The results show that for the nanotubes with specified length, the values of buckling strain and buckling mode parameter, experiences a decreasing trend with higher rate of changes with increasing the diameter of nanotube, which is same as the nanotubes with infinite length. As the length of structure increases the rate of change also increases and changes in the values of buckling strain get closer to each other at larger diameters. Furthermore, it can be seen that as the diameter of the structure increases, the changes in the values of buckling strain experiences a decreasing trend as the buckling mode parameter changes.

**Figure 5:**
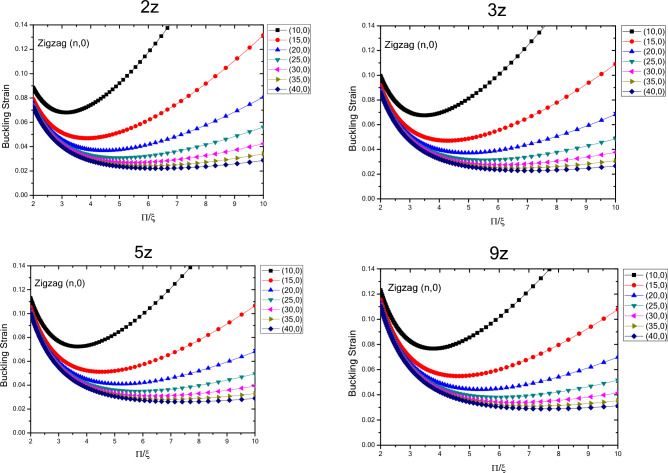
Changes in buckling strain values of zigzag nanotubes with specified lengths obtained by wrapping nanosheets with respect to inverse buckling mode parameter.

In Fig. 5, the variations in the buckling strain values of zigzag nanotubes with specified lengths obtained by wrapping zigzag nanosheets are shown in terms of the inverse buckling mode parameter. The results show that, same as the nanotubes with specified lengths obtained by wrapping armchair nanosheets and nanotubes with infinite length, by changing the buckling mode parameter, the buckling strain has a minimum value. In addition, it is seen that as the diameter of the structure increases, the variations in the buckling strain values become larger, and the changes in the buckling strain values get closer to each other for different nanotubes as the diameter of the nanotubes increases. In other words, the trend of changes in the buckling strain of nanotubes is similar to the buckling mode parameter changes in nanotubes with specified lengths and the ones with infinite lengths, but the quantum effects of fine scaling would result in of these values. Moreover, the results also show that by increasing the length of structure for the nanotubes obtained by wrapping zigzag nanosheets, the buckling strain increases and the magnitude of changes is higher in smaller diameters and compared to the nanotubes obtained by wrapped armchair nanosheets, the magnitude of the changes is larger. Furthermore, by increasing the length and diameter of the structure, the trend of change in the buckling strain with respect to the buckling mode parameter also decreases.

**Figure 6:**
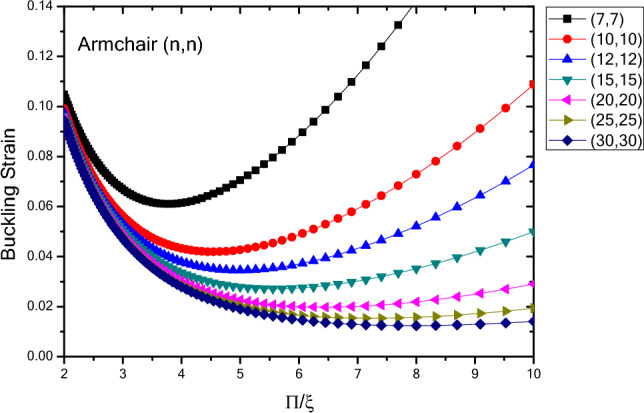
Buckling strain values $\left({\upvarepsilon }_{0}\right)$ of infinite length $\left(n,n\right)$ nanotubes with armchair atomic arrangement with respect to inverse buckling mode parameter $\xi$.

The buckling strain values ${(\upvarepsilon }_{0})$ with respect to the inverse mode modulus $(\xi )$ are plotted in Fig. 6 for nanotubes with infinite length, armchair atomic arrangement, and chirality of $\left(n,n\right)$. The results show that in the domain of the buckling mode parameter from small to large, the buckling strain values for the nanotubes with infinite length and armchair atomic arrangement, changes toward having a minimum value, which is the critical buckling strain. The smaller buckling mode parameter has a larger range of change in the buckling strain values and as the buckling mode parameter increases, changes occur in a smaller range. In addition, as the diameter of the nanotubes increases, the buckling strain values decrease and these values become closer for the nanotubes with larger diameters. The trend of buckling strain changes for nanotubes with infinite length and armchair atomic arrangement is similar to that of zigzag nanotubes, except that the buckling strain values of armchair nanotubes are in smaller domain with higher rate of change compared to zigzag nanotubes.

**Figure 7:**
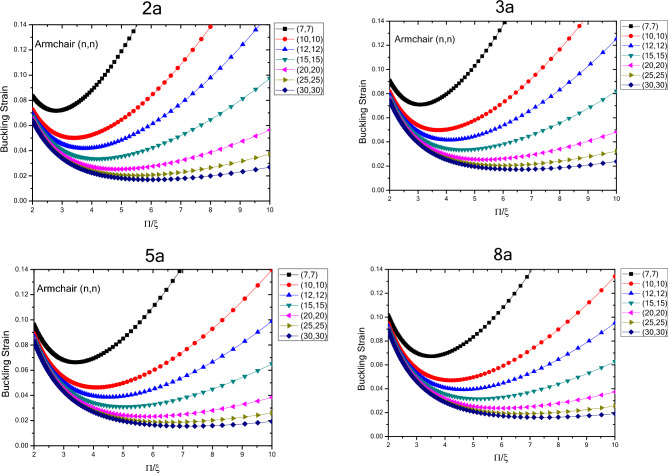
Changes of buckling strain values of armchair nanotubes with specified lengths obtained by wrapping armchair nanosheets with respect to inverse buckling mode parameter.

Figure 7 shows the variations in the buckling strain values with respect of the inverse mode buckling for armchair nanotubes with the specified lengths obtained by wrapping armchair nanosheets. The results show that by changing the buckling mode parameter, the nanotube buckling strain starts with changes in values close to each other in the larger buckling mode parameters and then when the buckling mode parameter decreases, it shows a wider range of changes which is similar to armchair nanotubes with infinite length. However, the quantum effects of the fine scaling and shape of the nanotubes have results in reduced buckling strain values as the length of the nanotube structure decreases. The range of buckling strain variations for different diameters in the larger buckling mode parameters varies over a larger range and as the length of the structure increases, the buckling strain values become closer to each other. It can be seen that as the length and diameter of the nanotubes increase, the buckling strain for a specific diameter, changes with lower slope. By comparing the changes in the quantities of the fine-scale armchair and zigzag nanotubes, it should be noted that the overall trend is similar. Except that the critical strain values of the armchair nanotubes are lower than the zigzag nanotubes and the quantum effects caused by fine scaling, is higher in nanotubes with armchair atomic arrangement compared to the ones with zigzag atomic arrangement.

**Figure 8:**
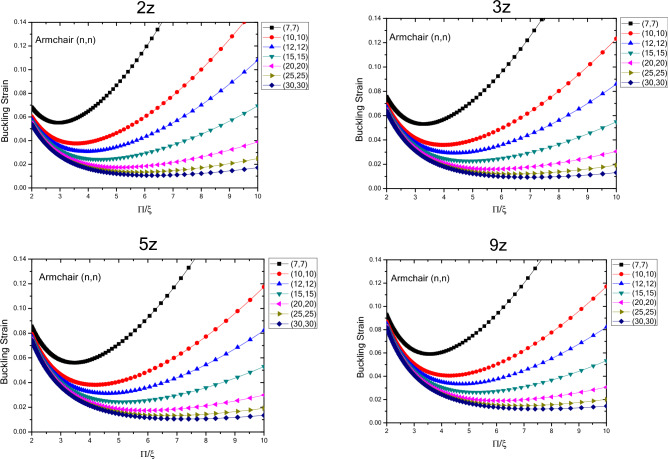
Changes in buckling strain values of armchair nanotubes with specified lengths obtained by wrapping zigzag nanosheets with respect to inverse buckling mode parameter.

The variations in the buckling strain values with respect to inversed buckling mode parameter are plotted in Fig. 8 for the armchair nanotubes with specified lengths obtained by wrapping zigzag nanosheets. The results show that as the length of the structure increases, the buckling strain has an increasing trend and these changes are greater for smaller lengths. According to the results, similar to infinite armchair nanotubes, as the buckling mode parameter changes from larger values to lower values, the buckling strain changes in a similar trend as it starts with values with less variation range and eventually the range of variation becomes wider. In addition, as the diameter of the nanotubes becomes larger in higher buckling mode parameters, the buckling strain values become closer to each other and as the parameter of buckling mode becomes smaller, the buckling strain values would have a wider range of variation. Compared to infinite armchair nanotubes, the buckling strain of armchair nanotubes obtained by wrapping zigzag nanosheets, has smaller values in the larger buckling mode parameters, and with the variations in the diameter and the buckling mode parameter, the buckling strain values have more variability. The graph also shows that by decreasing the buckling mode parameter, the variations of the buckling strain values are such that the larger buckling strain values are more on the side of the parameters with the smaller buckling mode. Compared to armchair nanotubes obtained by wrapping armchair nanosheets, the ones that are obtained by wrapping zigzag nanosheets have smaller buckling strain values, greater buckling strain values with respect to changes in diameter and larger buckling mode parameter. Compared to zigzag nanotubes obtained by wrapping zigzag nanosheets, it also has smaller buckling strain values, greater buckling strain values with respect to diameter and larger buckling mode parameter variations, and a larger slope of buckling strain values in smaller buckling mode parameters. The results show that with increasing diameter and length of structure, the changes of buckling strain with respect to buckling mode parameter have a decreasing trend.

**Figure 9:**
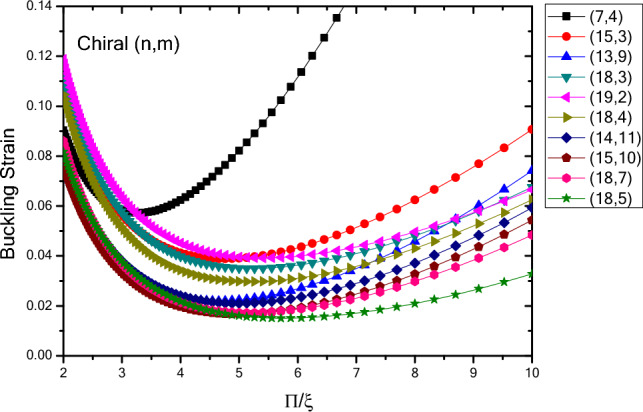
Changes in buckling strain values of infinite nanotubes with different chiralities in terms of inverse buckling mode parameter.

In Fig. 9, the variations in the buckling strain values of the infinite nanotubes with different chirality are plotted with respect to the inverse buckling mode parameter. It can be seen that the trend of changes in the buckling strain values is in a way that with changes in the larger buckling mode parameter, the buckling strain values start from values within a close range, and a similar trend and eventually in the larger buckling mode parameters they would have a wider range of values. In addition, as the diameter increases, there is also an increase in the range of the buckling strain values in the smaller buckling mode parameters. Moreover, it is found that the buckling strain values of infinite chiral nanotubes in the larger buckling mode parameters have smaller buckling strain values compared to the infinite zigzag nanotubes. In addition, the range of buckling strain values is such that in some smaller diameters, the buckling strain values are larger and in the larger diameters, the buckling strain values are lower, compared to armchair nanotubes. In the smaller buckling mode parameters, the chiral nanotubes with smaller diameters have wider range of values and in larger diameters it is reversed compared to the armchair and zigzag nanotubes. The results show that as the diameter of the structure increases, the slope of the buckling strain changes with respect to the buckling mode parameter experiences a decreasing trend. The trend of changes in the buckling strain values of chiral nanotubes, compared to armchair and zigzag nanotubes, are larger, in the higher buckling mode parameters, and smaller in the lower buckling mode parameters. Due to the different chirality of the these nanotubes, the changes of their buckling strain values with respect to the diameter changes are less regular compared to the zigzag and armchair nanotubes, but the trend of changes in buckling strain values with respect to the buckling mode parameter is similar.

**Figure 10:**
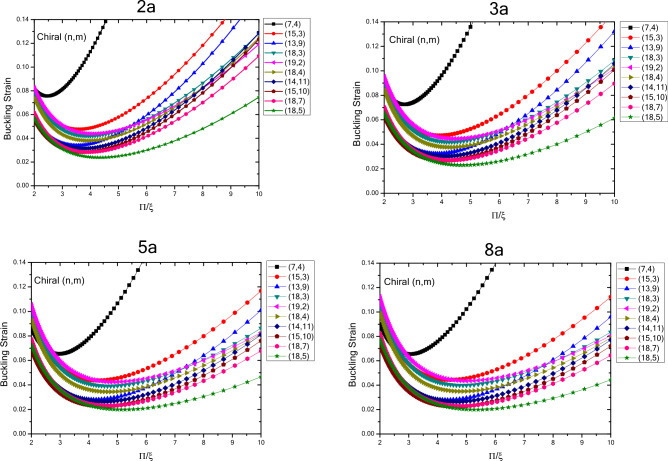
Changes of buckling strain values of specified lengths nanotubes with different chiralities obtained by wrapping armchair nanosheets with respect to inverse buckling mode parameter.

The variations in the buckling strain values with respect to inverse buckling mode parameter are plotted in Fig. 10 for nanotubes with different chirality and specified lengths obtained by wrapping armchair nanosheets. The results show that with increasing the length of the structure, the buckling strain values experiences an increasing trend, and this trend is reduced with increasing the length. Compared to chiral nanotubes with infinite length, the ones with specified length have smaller buckling strain values in larger buckling mode parameters that fall within the range of infinite nanotubes as the length of nanotubes increases. Moreover, the results show that as the diameter and length of the structure increase, the variation of the buckling strain with respect to the buckling mode parameter experiences a decreasing trend. In addition, the slope of graph in finite length nanotubes is higher in lower buckling mode parameters compared to nanotubes with infinite length. Minimum buckling strain values of finite length nanotubes increase from larger buckling mode parameters to smaller parameters as the length increases. Compared to Armchair and Zigzag nanotubes with finite length, the results show that the buckling strain values of different chiral nanotubes are smaller than the buckling strain values in larger buckling mode parameters for the finite length zigzag nanotubes and it is close to the values of armchair nanotubes with finite length. This trend continues at smaller buckling mode parameters except that at smaller buckling mode parameters the trend of change in the buckling strain values of the larger chiral nanotubes is higher.

**Figure 11:**
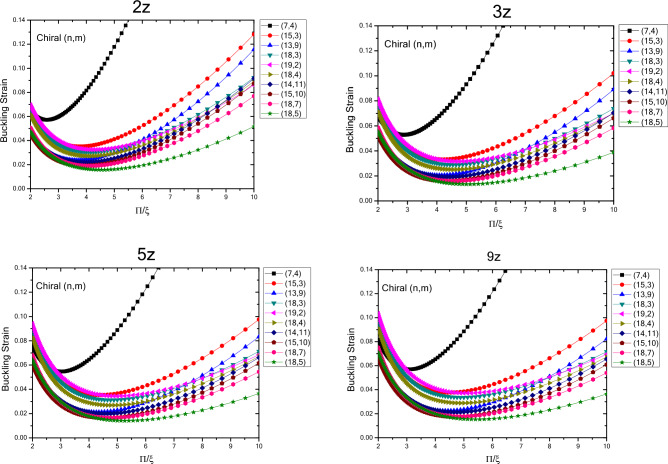
Changes of buckling strain values of nanotubes with different chiralities and specified lengths obtained by wrapping zigzag nanosheets with respect to inverse buckling mode parameter.

Figure 11 shows the variations in the buckling strain values with respect to the inverse of the buckling mode parameter for nanotubes with different chirality and specified lengths obtained by wrapping zigzag nanosheets. The results show that with increasing the length of the structure, the buckling strain values have an increasing trend and these changes decrease with increasing the length. Compared to infinite chiral nanotubes, chiral nanotubes with specified length have smaller buckling strain values in the larger buckling mode parameters, and compared to chiral nanotubes with specified length obtained by wrapping armchair nanosheets, the buckling strain values in the larger buckling mode parameters are in close range to each other. Compared to zigzag and armchair nanotubes with specified lengths obtained by wrapping zigzag nanosheets, the buckling strain values at the larger buckling mode parameters were close to the buckling strain values of the armchair nanotubes and smaller than the zigzag nanotubes obtained by wrapping zigzag nanosheets. Due to the chirality and the way the atoms are positioned in the structure of the chiral nanotubes, by varying the buckling mode parameter and the diameter of the nanotubes, their buckling strain values their buckling strain values are less regular compared to other nanotubes. Moreover, compared to other nanotubes, the buckling strain values in the smaller buckling mode parameters have a higher slope and the minimum buckling strain values are placed on the side of the smaller buckling mode parameters. Furthermore, as the diameter and length of the structure increase, the changes in the buckling strain with respect to the buckling mode parameter have a decreasing trend.

**Figure 12:**
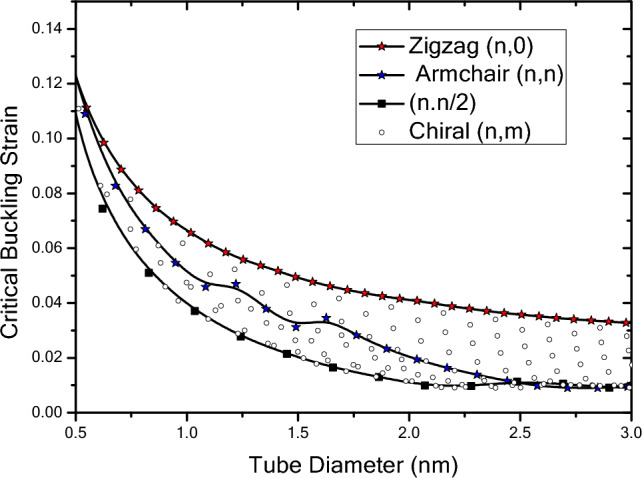
Critical buckling strain of infinite length nanotubes with respect to chirality and nanotube diameter.

In Fig. 12, the results of critical buckling strain analysis with respect to diameter of nanotube are presented for nanotubes with infinite length and different chiralites. Here, diameters up to 30 angstroms have been studied to investigate the quantum effects of fine scaling on the critical buckling strain of nanotubes. The results show that zigzag nanotubes with infinite length in the range of 5 to 30 angstroms have the highest critical buckling strain compared to nanotube with other chiralites. Critical buckling strain variations of zigzag nanotubes with infinite length have a uniform trend of change within the specified range for diameters due to their structural features and atomic arrangement. This uniform trend of change means that as the diameter of the nanotubes increases, their buckling strain values decrease and this trend is in a way that at diameters with the values less than 15 angstroms, larger changes would occur in the buckling strain compared to those with diameter values greater than 15 angstrom. In other words, as the diameter of the zigzag nanotubes increases, the buckling strain continues to decrease but the amount of these changes decreases, indicating that increasing the diameter reduces the resistance of the structure to the axial loading. In addition, at larger diameters where the quantum effects of fine scaling decrease, the trend of changes in the buckling strain also decreases, so some of the changes that lead to the reduction of the buckling strain can be attributed to the quantum effects of fine scaling. Moreover, the results show that the trend of change in the buckling strain of zigzag nanotubes with infinite length is always decreasing at the small diameters. For the armchair nanotubes with infinite lengths within the range of 5 to 30 angstroms, it can be seen that the critical buckling strain also shows a decreasing trend, except that the buckling strain of infinite armchair nanotubes in the range of 5 to 10 angstroms has different trend of changes compared to other diameters. In this range, the results of the critical buckling strain is closer to the results of zigzag nanotubes. In the range of 10 to 15 angstroms, the trend of changes for the critical buckling strain is different from the other ranges and the results are more distant from the critical buckling strain of the zigzag nanotubes. However, the results are closer to the critical buckling strain of nanotubes with chirality of $\left(n,n/2\right)$ than the zigzag nanotubes. The trend of change in the critical buckling strain results of the armchair nanotubes will change once more for diameters higher than 15 angstroms. As the diameter increases, the critical buckling strain results moves away from the results of zigzag nanotubes and gets more closer to nanotubes with chirality of $\left(n,n/2\right)$ and eventually from diameters higher than 25 angstroms, it is quite close to the results of nanotubes with chirality of $\left(n,n/2\right)$. The results also investigate the nanotubes with chirality of $\left(n,n/2\right)$ in the diameter range of 5 to 30 angstroms. It can be seen that the critical buckling strain of nanotubes with chirality of $\left(n,n/2\right)$ has a decreasing trend in the studied area and with increasing diameter, the reduction intensity of the critical buckling strain decreases. The results show that for diameters in the range of 5 to 10 angstroms, the buckling strain values of nanotubes with chirality of $\left(n,n/2\right)$ are closer to the results of armchair and zigzag nanotubes, and at larger diameters, it differs from the results of the armchair and zigzag nanotubes and the maximum spacing from zigzag nanotubes is reached in 30 angstroms. However, at the diameters larger than 25 angstroms, the results are close to the results of the armchair nanotubes. In general, as the diagram shows, the critical buckling strain of nanotubes with different chirality are obtained between the results of zigzag nanotubes and nanotubes with chirality of $\left(n,n/2\right)$. The results show that at very small diameters the buckling strain values of the nanotubes with different chirality are closer to each other. This may be due to the very small diameters of the nanotubes, and the type of buckling that takes place. Then, at slightly larger diameters, the trend is such that the buckling strain of nanotubes with different chirality differs markedly, and this area is where the chirality and arrangement of structural atoms have a greater effect on the buckling of nanotubes with different chirality. Subsequently, at larger diameters, that the structure of nanotubes slowly become closer to the structure of their constituent sheet, with slight increase in the diameter the critical buckling strain of the nanotubes with different chiralities become close to each other and the buckling occurs closer to the buckling of the sheet.

**Figure 13:**
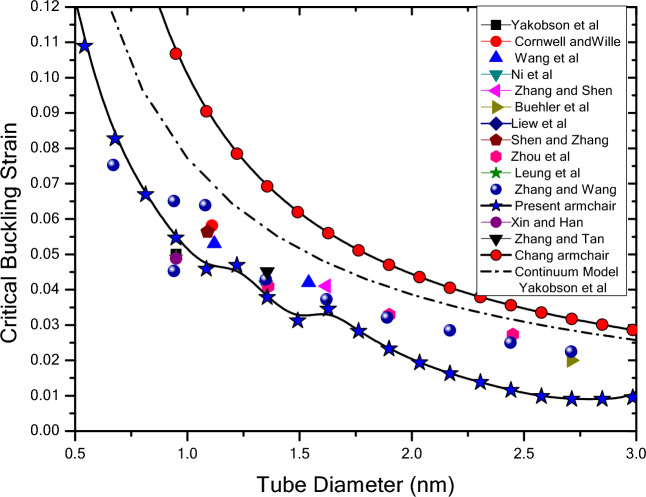
Variation of critical buckling strain versus diameter of nanotube obtained by various researchers using atomistic simulations under different simulation conditions.

Figure 13 shows the variation of critical buckling strain versus carbon nanotube diameter for the state of armchair. Also obtained results from Chang’s molecular mechanic^12^ and Yakobson’s continuum method^9,20^ are presented in Fig. 13 which shows large difference with those of molecular dynamic results. It can be seen that Chang’s model^12^ tends to overestimate the buckling strains, especially when the tube diameter decreases. In contrast, the results obtained from the present analysis are found to be in excellent agreement with the ones from the molecular dynamic simulations, which indicates the capability of the present approach in predicting buckling strains of carbon nanotubes.

Table 3 is provided to show the critical buckling strains of single-walled CNTs as obtained by various researchers using atomistic simulations under different simulation conditions. It’s clear that the obtained results in this study is in good agreement with those obtained by molecular dynamic method in comparison with continuum method and molecular mechanics done ever before^9,10,51,57,98–106^. Yakobson et al.^9^ obtained a critical buckling strain of 5% for a (7, 7) tube from classical molecular dynamics (MD). This value is much lower than Chang’s prediction of about 10.7% from molecular mechanics^12^ and the prediction by Yakobson’s continuum model of 7.7%^9,20^ and what obtained in present prediction for critical buckling strain is 6.1% that has less difference with Yakobson’s results in comparison with Yakobson’s continuum model and Chang’s molecular mechanics. For (10,10) tube the Chang’s prediction is 6.92% and Yakobson’s continuum model prediction is 5.69% which is more than Zhang’s^104^ results obtained by molecular dynamics (MD) which is 4.29% and the obtained critical buckling strain in this prediction is 4.23%. Differences between Chang’s molecular mechanics and present molecular mechanics method are due to technique of acquisition of energy equation’s terms. The present approach gives the total potential energy without any simplification.

**Table 3:** Critical strains of single-walled CNTs as obtained by various researchers using atomistic simulations under different simulation conditions.

Authors	Potential	Simulation conditions	Chiral indices	*d* (nm)	Critical strain
Yakobson et al.^9^	Tersoff–Brenner (TB)	Conjugate gradient	(7, 7)	0.95	0.05
Cornwell et al.^98^	TB	0.005 K by velocity scaling	(8, 8)	1.11	0.0581
			(16, 16)	2.22	0.0381
			(19, 19)	2.63	0.0312
			(24,24)	3.33	0.0256
Wang et al.^99^	REBO	0.01 K by Nose–Hoover thermostat	(8, 8)	1.12	0.053
			(11, 11)	1.54	0.042
			(17, 17)	2.38	0.032
			(23, 23)	3.20	0.0225
Ni et al.^57^	REBO	100 K by Langevin thermostat	(10, 10)	1.35	0.0414
Zhang et al.^10^	REBO	300 K by Nose–Hoover thermostat	(12, 12)	1.62	0.041
Buehler et al.^51^	TB	×	(20, 20)	2.71	0.02
Liew et al.^100^	REBO	Conjugate gradient	(10, 10)	1.36	0.067
Shen et al.^101^	REBO	300 K by Nose–Hoover thermostat	(8, 8)	1.09	0.0564
Zhou et al.^102^	TB	100 K by Berendsen thermostat	(10, 10)	1.36	0.0409
			(14, 14)	1.90	0.0329
			(18, 18)	2.45	0.0273
Leung et al.^103^	REBO	Atomic scale finite element method	(7, 7)	0.95	0.0492
Zhang et al.^104^	REBO	300 K by velocity scaling	(5, 5)	0.67813	0.0753
			(7, 7)	0.94939	0.0651
			(7, 7)	0.94939	0.0453
			(8, 8)	0.0639	0.0639
			(10, 10)	1.35627	0.0429
			(12, 12)	1.62752	0.0373
			(14, 14)	1.89878	0.0321
			(16, 16)	2.17003	0.0285
			(18, 18)	2.44128	0.0250
			(20, 20)	0.0225	0.0225
Xin et al.^105^	Morse, harmonic and periodic	Strain rates $\dot{\upvarepsilon }=2.5\times {10}^{-3}\frac{nm}{ps}$	(7, 7)	0.95	0.0488
Zhang et al.^106^	REBO	Strain rates $\dot{\upvarepsilon }=8.3\times {10}^{-4}\frac{nm}{ps}$	(10, 10)	1.35627	0.0451
		Strain rates $\dot{\upvarepsilon }=8.3\times {10}^{-5}\frac{nm}{ps}$	(10, 10)	1.35627	0.0544
Present authors	MM		(3, 3)	0.40688	0.15007
			(4, 4)	0.54251	0.10892
			(5, 5)	0.67813	0.08274
			(6, 6)	0.81376	0.0669
			(7, 7)	0.94939	0.05464
			(8, 8)	1.08502	0.04583
			(9, 9)	1.22064	0.0469
			(10, 10)	1.35627	0.03786
			(11, 11)	1.4919	0.03121
			(12, 12)	1.62752	0.03454
			(13, 13)	1.76315	0.02825
			(14, 14)	1.89878	0.02328
			(15, 15)	2.0344	0.01935
			(16, 16)	2.17003	0.01626
			(17, 17)	2.30566	0.01379
			(18, 18)	2.44128	0.01157
			(19, 19)	2.57691	0.0098
			(20, 20)	2.71254	0.00903
			(21, 21)	2.84817	0.00901
			(22, 22)	2.98379	0.00946
			(23, 23)	3.11942	0.01693
			(24, 24)	3.25505	0.01402
			(25, 25)	3.39067	0.01146

To investigate the influence of the chirality on the buckling strains of nanotubes, the variation of critical buckling strain versus diameter of nanotube is plotted in Fig. 13 for armchair, zigzag and chiral tubes. The difference between results obtained for armchair and zigzag tubes becomes more pronounced when the diameter of tube increases. As it can be seen in this figure, the stability of zigzag carbon nanotube under axial load is better than armchair carbon nanotube and the minimum stability is for $\left(n,n/2\right)$ tube. Furthermore, this figure presents the critical buckling strain of chiral nanotube in which the variation of critical buckling strain in chiral tube is such that for a certain diameter, increasing in the chiral angle from $\theta =0$ to $\theta =\pi /12$, critical buckling strain changes from zigzag to $\left(n,n/2\right)$ tube. With continuing the trend of increase in chiral angle from $\theta =\pi /12$ to $\theta =\pi /6$, critical buckling strain changes from ($n,n/2$) tube to ($n,n$) tube.

**Figure 14:**
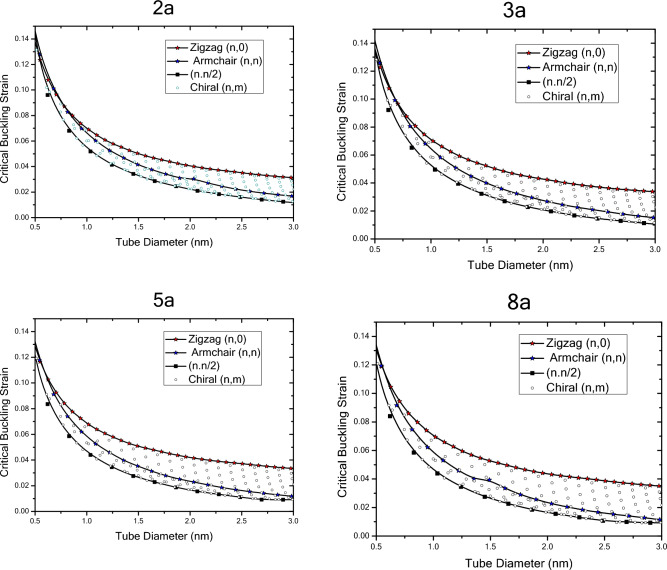
Critical buckling strain of finite length nanotubes obtained by wrapping armchair nanosheets with respect to chirality and nanotube diameter.

Figure 14 shows the results of the critical buckling strain with respect to nanotube diameter for finite length nanotubes with different chiralities obtained by wrapping armchair nanosheets. The critical buckling strain variations of finite length nanotubes are in a trend similar to the critical buckling strain of nanotubes with infinite length. As the results show, in general, the changes are in a way that the critical buckling strain decreases with increasing the diameter of the structure, which depends on the length of structure, diameter and the arrangement of the atoms. In all nanotubes of different lengths, the critical buckling strain of the zigzag nanotubes has a decreasing trend with respect to the diameter, and with increasing structure length, the critical buckling strain of the zigzag nanotubes has a decreasing trend compared to the shorter lengths. By increasing the length of zigzag nanotubes, the trend of change in their critical buckling strain decreases, so that for shorter lengths and for diameters of lower than 15 angstroms, the critical buckling strain variations are greater. For armchair nanotubes, as the length of the structure decreases, the critical buckling strain decreases and by increasing the length of the structure, the values of the critical buckling strain decrease at a specified diameter. At shorter lengths, the critical buckling strain of armchair nanotubes in the larger diameter range is closer to the critical buckling strain of the zigzag nanotubes and by increasing the length of the nanotubes at smaller diameters, it deviates from the critical buckling strain of the zigzag nanotubes. The critical buckling strain of the armchair nanotubes at smaller lengths, even if it has a large diameter, is still smaller than the critical buckling strain of nanotubes with the chirality of $\left(n,n/2\right)$. However, as nanotubes grow larger in diameter, they deviate from the results of zigzag nanotubes and become closer to the results of $\left(n,n/2\right)$ nanotubes. The buckling strain of the armchair nanotubes in smaller lengths has uniform decreasing trend and as the length increases, the intensity of this trend and the critical buckling strain changes would be different from the variations in smaller lengths. It can be seen that for $\left(n,n/2\right)$ nanotubes the critical buckling strain has a similar trend to the infinite nanotubes and compared to the ones with infinite length, the trend of changes and the decrease in critical strain values reduces as the length increases. Moreover, the critical strain values for a specified diameter experiences a decreasing trend as the length of nanotube increases. Comparison of the critical buckling strain results of the finite and infinite length nanotubes reveals that the as the length the diameter of the nanotubes become smaller, the critical strain values of the nanotubes with different chirality become closer to each other. In addition, at larger lengths and larger diameters, chiral differentiation becomes more evident on the trend of changes of critical buckling strain, insofar as increasing the diameter does not eliminate the curvature effects of the nanotubes.

**Figure 15:**
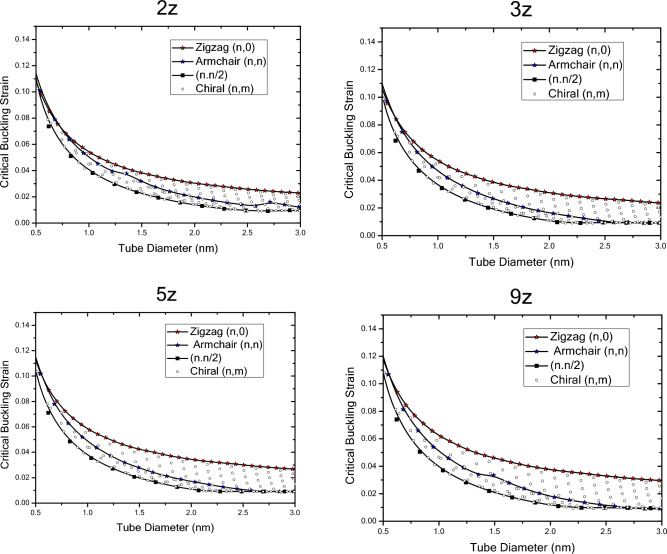
Critical buckling strain of finite length nanotubes obtained by wrapping zigzag nanosheets with respect to chirality and nanotube diameter.

In Fig. 15, results of the critical buckling strain with respect to nanotube diameter of finite-length nanotubes obtained by wrapping zigzag nanosheets are plotted. The results show that the general trend of buckling strain variations of nanotubes with different chiralities obtained by wrapping zigzag nanosheets is similar to finite and infinite nanotubes obtained by wrapping armchair nanosheets in a way that with increasing the diameter of the structure, the critical buckling strain decreases. However, the variations in their critical buckling strain values are different. The buckling strain variations of finite zigzag nanotubes obtained by wrapping zigzag nanosheets initially begin with values lower than the critical buckling strain values of zigzag nanotubes with infinite length and zigzag nanotubes obtained by wrapping armchair nanosheets and as the diameter increases, it experiences a decreasing trend. In addition, with increasing length, the buckling strain values increases in specific diameter until it reach the values of infinite nanotubes. The slope of the critical buckling strain variations at smaller diameters is greater than that of the larger diameters, and as the diameter changes, there is a regular decreasing trend in the values. The trend of changes in the critical buckling strain values of zigzag nanotubes obtained by wrapping zigzag nanosheets is lower than that of infinite and finite zigzag nanotubes obtained by wrapping armchair nanosheets. The results show that the critical buckling strain of nanotubes obtained by wrapping zigzag nanosheets changes with increasing diameter in a way that, at shorter lengths, the values are smaller than that of infinite nanotubes and armchair nanotubes obtained by wrapping armchair nanosheets with similar diameters. In addition, with increasing length, it approaches critical buckling strain values of nanotubes with infinite length. As the diameter increases, the buckling strain of the armchair nanotubes are initially closer to the critical buckling strain values of the zigzag nanotubes and as the diameter increases, the changes are such that they approach the critical strain of$\left(n,n/2\right)$ nanotubes. Moreover, the results show that the critical buckling strain of $\left(n,n/2\right)$ nanotubes obtained by wrapping zigzag nanosheets, starts with a similar trend of change and lower values than infinite $\left(n,n/2\right)$ nanotubes obtained by wrapping armchair nanosheets which decreases with increasing diameter and with increasing length, it continius to enlarge until it reaches the value of infinite nanotubes.

In Table 4, the critical buckling strain results of zigzag nanotubes with finite and infinite lengths obtained by wrapping armchair and zigzag nanosheets are presented. In order to study and quantify the critical buckling strain changes of the nanotubes, the percentage of their critical buckling strain changes has also been studied. To compare the results, zigzag nanotubes with diameter from about 2 to 30 angstroms were studied. The results show that in zigzag nanotubes with infinite length, critical buckling strain decreases with increasing diameter, such that in smaller diameters the trend of changes is higher than the changes in larger diameters and in larger diameters the trend of change in the critical buckling strain is reduced due to the reduction of nanotube curvature. The results show that at the diameters less than 6 angstroms, the percentage of buckling strain changes is greater than the percentage of diameter changes, which then decreases. The results show that the ratio of critical buckling strain variations to diameter changes in diameters larger than 25 angstroms is less than one. This implies that in finite length nanotubes, the buckling strain is more dependent on the diameter changes and because of the small length of the structure, the quantum effects caused by the changes in the diameter to the larger diameters are even more significant. Results for zigzag nanotubes obtained by wrapping 3a armchair nanosheets show that the buckling strain variations compared to nanotubes with shorter length are lower, and these variations are higher in larger nanotubes with larger lengths. The results also show that the ratio of critical buckling strain to nanotube diameter changes at diameters below 15 angstroms is higher than one, which decreases for larger diameters. Moreover, the results show that with increasing length of zigzag nanotubes obtained by wrapping armchair nanosheets, the critical buckling strain variations at smaller diameters decrease and it gets closer to the variations of nanotubes with infinite lengths. In addition, as the diameter increases, trend of changes of the critical buckling strain reduces and eventually approaches the trend of changes of the critical buckling strain of the nanotubes with infinite length. The ratio of the critical buckling strain changes to nanotube diameter changes in zigzag nanotubes obtained by wrapping 5a and 8a armchair nanosheets at diameters less than 10 angstroms is higher than one and it is reduced at larger diameters. For zigzag nanotubes obtained by wrapping 2z zigzag nanosheets, the results show that the critical buckling strain at larger diameters has more variations compared to diameter changes and with increasing the diameter the trend of changes decreases. Compared to infinite length nanotubes, the critical buckling strain variations are greater and compared to the finite length nanotubes obtained by wrapping 2a armchair nanosheets at smaller diameters they have smaller buckling strain variations, which is reversed at larger diameters. The ratio of critical buckling strain variations to nanotube diameters, in diameter less than 25 angstroms is higher than one which decreases in larger diameters. For zigzag nanotubes obtained by wrapping 3z zigzag nanosheets, the buckling strain variations are smaller than those of with shorter length and nanotubes obtained by wrapping 3a armchair nanosheets, and are closer to the variation of infinite length nanotubes. Furthermore, the results show that the critical buckling strain variations of zigzag nanotubes obtained by wrapping zigzag nanosheets are lower than zigzag nanotubes obtained by wrapping armchair nanosheets at smaller diameters, which increases at larger diameters. In addition, with increasing the length of the nanotube structure, due to the nature of the sheets which are wrapped to form theses nanotubes, the critical buckling strain changes occur from lower to higher in smaller diameters. By increasing the length of zigzag nanotubes obtained by wrapping zigzag nanosheets, the trend of changes of the critical buckling strain reduces with respect to the changes of the diameter. In addition, the ratio of the critical buckling strain to the diameter changes of the nanotubes in the zigzag nanotubes obtained by wrapping 5z and 9z zigzag nanosheets is larger than one in diameters lower than 10 angstroms, which reduces in larger diameters.

**Table 4:** Critical buckling strain results of zigzag nanotubes with finite and infinite lengths obtained by wrapping armchair and zigzag nanosheets.

	Chirality	(3, 0)	(4, 0)	(5, 0)	(6, 0)	(7, 0)	(12, 0)	(20, 0)	(26, 0)	(32, 0)	(40, 0)
	Diameter	0.2349	0.3132	0.3915	0.4698	0.5481	0.9397	1.5661	2.0359	2.5057	3.1322
	Variation percentage	0	33.336	66.668	100	133.34	300	566.67	766.68	966.68	1233.3
Infinite length	Critical strain	0.2781	0.1979	0.1558	0.1294	0.1113	0.0697	0.0477	0.0407	0.0357	0.0321
	Variation percentage	0	− 40.536	− 78.57	− 114.9	− 149.9	− 299.1	− 483.4	− 582.67	− 679	− 766.7
	Ratio of strain changes to diameter changes		− 1.216	− 1.178	− 1.149	− 1.124	− 0.997	− 0.853	− 0.76	− 0.702	− 0.622
2a	Critical strain	0.3871	0.2422	0.1795	0.1458	0.1236	0.0737	0.0485	0.04	0.0351	0.0304
	Length to diameter ratio ($L/D$)	31.43044	23.57283	18.85826	15.71522	13.47019	7.856773	4.714265	3.626411	2.946486	2.357132
	Variation percentage	0	− 59.826	− 115.7	− 165.5	− 213.1	− 425.2	− 699	− 867.34	− 1003	− 1173
	Ratio of strain changes to diameter changes		− 1.7946	− 1.735	− 1.655	− 1.598	− 1.417	− 1.234	− 1.1313	− 1.038	− 0.951
3a	Critical strain	0.3484	0.2288	0.1753	0.1438	0.1227	0.0748	0.0504	0.0424	0.037	0.0329
	Length to diameter ratio ($L/D$)	52.38395	39.28796	31.43037	26.19198	22.45026	13.09459	7.857091	6.044005	4.910799	3.928545
	Variation percentage	0	− 52.273	− 98.71	− 142.4	− 184	− 366	− 592	− 722.28	− 842.6	− 958
	Ratio of strain changes to diameter changes		− 1.568	− 1.481	− 1.424	− 1.38	− 1.22	− 1.045	− 0.9421	− 0.872	− 0.777
5a	Critical strain	0.3054	0.2113	0.1643	0.1358	0.1165	0.0725	0.0493	0.0415	0.0372	0.0327
	Length to diameter ratio ($L/D$)	94.29132	70.71849	56.57479	47.14566	40.41056	23.57032	14.14279	10.87923	8.839458	7.071397
	Variation percentage	0	− 44.54	− 85.86	− 124.9	− 162.2	− 321.4	− 518.9	− 635.83	− 721.3	− 835
	Ratio of strain changes to diameter changes		− 1.3361	− 1.288	− 1.248	− 1.217	− 1.071	− 0.916	− 0.8293	− 0.746	− 0.677
8a	Critical strain	0.3044	0.213	0.1663	0.1378	0.1188	0.0741	0.0513	0.0432	0.0386	0.0342
	Length to diameter ratio ($L/D$)	157.1519	117.8639	94.29114	78.57595	67.35081	39.28379	23.57128	18.13202	14.7324	11.78564
	Variation percentage	0	− 42.87	− 82.98	− 120.9	− 156.2	− 311	− 493.7	− 604.89	− 688.1	− 788.9
	Ratio of strain changes to diameter changes		− 1.286	− 1.245	− 1.209	− 1.171	− 1.037	− 0.871	− 0.789	− 0.712	− 0.64
2z	Critical strain	0.2923	0.1874	0.1414	0.1151	0.0975	0.0575	0.037	0.03	0.0257	0.0223
	Length to diameter ratio ($L/D$)	30.24398	22.68298	18.14639	15.12199	12.9617	7.560189	4.536307	3.489518	2.83526	2.268153
	Variation percentage	0	− 55.994	− 106.7	− 154	− 199.7	− 408.1	− 689.9	− 873.91	− 1035	− 1213
	Ratio of strain changes to diameter changes		− 1.6797	− 1.6	− 1.54	− 1.498	− 1.36	− 1.217	− 1.1399	− 1.071	− 0.983
3z	Critical strain	0.2614	0.1777	0.1371	0.1123	0.0957	0.0573	0.0372	0.0306	0.0266	0.0229
	Length to diameter ratio ($L/D$)	48.39034	36.29275	29.0342	24.19517	20.73872	12.0963	7.258087	5.583226	4.536413	3.629043
	Variation percentage	0	− 47.104	− 90.65	− 132.7	− 173.1	− 356.1	− 602.4	− 755.52	− 884	− 1044
	Ratio of strain changes to diameter changes		− 1.413	− 1.36	− 1.327	− 1.298	− 1.187	− 1.063	− 0.9854	− 0.915	− 0.846
5z	Critical strain	0.2659	0.1851	0.1441	0.1187	0.1016	0.0618	0.0412	0.034	0.03	0.026
	Length to diameter ratio ($L/D$)	84.6828	63.5121	50.80968	42.3414	36.29263	21.16845	12.70161	9.770613	7.938696	6.350805
	Variation percentage	0	− 43.668	− 84.52	− 124	− 161.9	− 330.6	− 545.5	− 681.69	− 785.5	− 921.6
	Ratio of strain changes to diameter changes		− 1.3099	− 1.268	− 1.24	− 1.214	− 1.102	− 0.963	− 0.8891	− 0.813	− 0.747
9z	Critical strain	0.274	0.1928	0.1502	0.1241	0.1067	0.0656	0.0447	0.0372	0.0328	0.0289
	Length to diameter ratio ($L/D$)	157.2685	117.9513	94.36107	78.63423	67.40077	39.31293	23.58876	18.14547	14.74333	11.79438
	Variation percentage	0	− 42.14	− 82.47	− 120.7	− 156.7	− 317.5	− 513.1	− 636.73	− 734.6	− 848.1
	Ratio of strain changes to diameter changes		− 1.2641	− 1.237	− 1.207	− 1.175	− 1.058	− 0.905	− 0.8305	− 0.76	− 0.688

The critical buckling strain results of armchair nanotubes with infinite and finite lengths obtained by wrapping armchair and zigzag nanotubes are presented in Table 5. The results show that with increasing the diameter of nanotubes, the critical buckling strain has a decreasing trend. For this study the diameters in the range of about 2 to 30 angstroms are chosen for armchair nanotubes. In the infinite armchair nanotubes, with increasing the diameter, the critical buckling strain reduces and as the diameter increases, the trend of changes also increases in a way that the ratio of critical buckling strain to nanotube diameter changes has always been greater than one and increases until the diameter reaches the value of 25 angstroms. Compared to zigzag nanotubes with infinite length, the critical buckling strain variations were higher for the armchair nanotubes, and this increase in the magnitude of the variations with respect to changes in the diameter continues up to larger diameters. For the finite length armchair nanotubes obtained by wrapping 2a armchair nanosheets, the results show that with increasing diameter the critical buckling strain structure of the nanotubes has a decreasing trend. In addition, compared to the infinite length armchair nanotubes, it can be seen that in the critical buckling strain starts at larger values in the smaller nanotubes and in the larger diameters the critical buckling strain of the smaller length nanotubes is greater. Then, with increasing the diameter, for diameters larger than 15 angstroms, the critical buckling strain variations of the nanotubes with infinite length are increased. Compared to similar zigzag nanotubes with finite length, the critical buckling strain with respect to similar diameters have lower values and would experience greater variations with respect to diameter changes. The ratio of critical buckling strain to nanotube diameter changes has always been greater than one, but with increasing the diameter, unlike zigzag, armchair, and infinite length nanotubes, it experiences a decreasing trend, which is reversed at diameters greater than 20 angstroms. For limited length armchair nanotubes obtained by wrapping 3a armchair nanosheets the critical buckling strain has a decreasing trend with increasing diameter, and the buckling strain values are higher than those of nanotubes with infinite length and lower than the smaller length nanotubes. Critical buckling strain changes have an increasing trend and it has fewer changes compared to nanotubes with shorter length. The ratio of critical buckling strain to nanotube diameter changes has always been greater than one and in the diameters less than 10 angstroms, it has a decreasing trend. Compared to zigzag nanotubes made from similar nanosheets, they have smaller buckling strain and more variations. For armchair nanotubes obtained by wrapping 5a and 8a armchair nanosheets, the critical buckling strain has a decreasing trend and the values of the critical buckling strain reduces with increasing the lengths and compared to similar zigzag nanotubes they have less buckling strain and more variation as the diameter changes. The ratio of critical buckling strain to nanotube diameter changes is always greater than one and in the diameters less than 7 angstroms, it has a decreasing trend. For the finite length armchair nanotubes obtained by wrapping 2z zigzag nanosheets, the results show that as the diameter increases the critical buckling strain decreases and the buckling strain values are lower compared to the infinite length nanotubes have greater variations with increasing diameter. Compared to armchair nanotubes obtained by wrapping similar armchair nanosheets, they had lower critical buckling strain values and lower variations. Compared to zigzag nanotubes obtained by wrapping zigzag nanosheets, they have a lower critical buckling strain but have larger diameter-dependent variations. The ratio of critical buckling strain to nanotube diameter changes is always greater than one and in the diameters smaller than 7 angstroms, it has a decreasing trend and higher than 7 angstroms, it has an increasing trend. For armchair nanotubes obtained by wrapping 3z zigzag nanosheets the critical buckling strain has a decreasing trend and its values are lower than armchair nanotubes obtained by wrapping armchair nanosheets and smaller diameter-dependent variation. Compared to zigzag nanotubes obtained by wrapping wrapped zigzag nanosheets, they have lower critical buckling strain and larger diameter-dependent changes. The ratio of critical buckling strain to nanotube diameter changes has always been greater than one and in diameters smaller than 5 angstroms, it has a decreasing trend, which is reversed in diameters higher than 5 angstroms. For armchair nanotubes obtained by wrapping 5z and 9z zigzag nanosheets, the critical buckling strain has a decreasing trend and compared to the infinite length nanotubes they have lower critical buckling strain values, which increases with increasing length. Compared to zigzag nanotubes obtained by wrapping zigzag nanosheets, they also have lower critical buckling strain and higher diameter-dependent changes.

**Table 5:** Critical buckling strain results of armchair nanotubes with finite and infinite lengths obtained by wrapping armchair and zigzag nanosheets.

	Chirality	(2, 2)	(3, 3)	(4, 4)	(5, 5)	(6, 6)	(8, 8)	(11, 11)	(15, 15)	(18, 18)	(23, 23)
	Diameter	0.2713	0.4069	0.5425	0.6781	0.8138	1.085	1.4919	2.0344	2.4413	3.1194
	Variation percentage	0	50.002	100	150	200	300.01	450.01	650.01	800.01	1050
Infinite length	Critical strain	0.2378	0.1501	0.1089	0.0827	0.0669	0.0458	0.0312	0.0194	0.0116	0.0169
	Variation percentage	0	− 58.46	− 118.33	− 187.4	− 255.46	− 418.9	− 661.9	− 1129	− 1955	− 1304.6
	Ratio of strain changes to diameter changes		− 1.169	− 1.1832	− 1.249	− 1.2773	− 1.396	− 1.471	− 1.737	− 2.444	− 1.2425
2a	Critical strain	0.3456	0.181	0.1279	0.1011	0.0827	0.0603	0.0415	0.0303	0.0224	0.0156
	Length to diameter ratio ($L/D$)	27.21345	18.14453	13.60924	10.88779	9.072266	6.804618	4.94873	3.629085	3.024213	2.366805
	Variation percentage	0	− 90.98	− 170.29	− 241.8	− 317.73	− 473.4	− 731.9	− 1040	− 1446	− 2113.9
	Ratio of strain changes to diameter changes		− 1.819	− 1.7028	− 1.612	− 1.5886	− 1.578	− 1.627	− 1.6	− 1.808	− 2.0132
3a	Critical strain	0.3078	0.1723	0.1258	0.0992	0.0805	0.0582	0.04	0.0268	0.021	0.0142
	Length to diameter ratio ($L/D$)	45.35566	30.24082	22.68201	18.14628	15.12041	11.341	8.247865	6.048461	5.040343	3.944666
	Variation percentage	0	− 78.62	− 144.63	− 210.2	− 282.16	− 428.6	− 669.7	− 1048	− 1366	− 2064.5
	Ratio of strain changes to diameter changes		− 1.572	− 1.4462	− 1.402	− 1.4108	− 1.429	− 1.488	− 1.612	− 1.708	− 1.9661
5a	Critical strain	0.2661	0.1602	0.1177	0.0911	0.074	0.0526	0.0354	0.0229	0.0169	0.0108
	Length to diameter ratio ($L/D$)	81.64036	54.4336	40.82771	32.66337	27.2168	20.41385	14.84619	10.88725	9.072638	7.100414
	Variation percentage	0	− 66.13	− 126.02	− 192.2	− 259.57	− 406.3	− 652	− 1062	− 1473	− 2368.6
	Ratio of strain changes to diameter changes		− 1.323	− 1.2602	− 1.282	− 1.2978	− 1.354	− 1.449	− 1.633	− 1.841	− 2.2558
8a	Critical strain	0.2642	0.162	0.1194	0.0916	0.0747	0.053	0.0393	0.0227	0.0167	0.0104
	Length to diameter ratio ($L/D$)	136.067	90.72249	68.04605	54.43884	45.36124	34.02302	24.7436	18.14539	15.12103	11.834
	Variation percentage	0	− 63.07	− 121.17	− 188.3	− 253.77	− 398.4	− 571.7	− 1065	− 1480	− 2447.3
	Ratio of strain changes to diameter changes		− 1.261	− 1.2116	− 1.255	− 1.2688	− 1.328	− 1.27	− 1.638	− 1.85	− 2.3308
2z	Critical strain	0.2591	0.1401	0.1004	0.0789	0.0636	0.0452	0.0319	0.0193	0.0141	0.0106
	Length to diameter ratio ($L/D$)	26.18618	17.4596	13.0955	10.47679	8.729798	6.547751	4.761921	3.492091	2.910052	2.27746
	Variation percentage	0	− 84.99	− 158.1	− 228.4	− 307.23	− 473.6	− 711.7	− 1242	− 1737	− 2337.3
	Ratio of strain changes to diameter changes		− 1.7	− 1.5809	− 1.523	− 1.5361	− 1.579	− 1.581	− 1.91	− 2.172	− 2.2259
3z	Critical strain	0.2279	0.1337	0.0973	0.0749	0.0603	0.0418	0.0268	0.016	0.011	0.0091
	Length to diameter ratio ($L/D$)	41.89786	27.93534	20.95279	16.76285	13.96767	10.4764	7.61907	5.587343	4.656081	3.643935
	Variation percentage	0	− 70.42	− 134.2	− 204.2	− 278.01	− 445.9	− 749.1	− 1323	− 1964	− 2418.2
	Ratio of strain changes to diameter changes		− 1.408	− 1.342	− 1.361	− 1.39	− 1.486	− 1.665	− 2.036	− 2.455	− 2.303
5z	Critical strain	0.2302	0.1397	0.1022	0.0779	0.0628	0.0435	0.028	0.0166	0.0112	0.0092
	Length to diameter ratio ($L/D$)	73.32101	48.88668	36.66726	29.33489	24.44334	18.33363	13.33333	9.777817	8.148114	6.376864
	Variation percentage	0	− 64.8	− 125.23	− 195.5	− 266.53	− 428.8	− 722.4	− 1283	− 1952	− 2402
	Ratio of strain changes to diameter changes		− 1.296	− 1.2522	− 1.303	− 1.3326	− 1.429	− 1.605	− 1.974	− 2.439	− 2.2875
9z	Critical strain	0.2365	0.1458	0.1068	0.0814	0.0659	0.0458	0.0334	0.0175	0.0121	0.009
	Length to diameter ratio ($L/D$)	136.1679	90.78978	68.09652	54.47922	45.39489	34.04826	24.76195	18.15885	15.13225	11.84278
	Variation percentage	0	− 62.19	− 121.4	− 190.7	− 258.91	− 416.5	− 608.8	− 1248	− 1851	− 2515.6
	Ratio of strain changes to diameter changes		− 1.244	− 1.2139	− 1.271	− 1.2945	− 1.388	− 1.353	− 1.92	− 2.314	− 2.3958

In Table 6, the critical buckling strain results of $\left(n,n/2\right)$ nanotubes, which is a chirality between zigzag nanotubes with chirality of $\left(n,0\right)$ and armchair nanotubes with chirality of $\left(n,n\right)$ structures, are compared with the finite and infinite lengths nanotubes obtained by wrapping armchair and zigzag nanosheets. Same as other structures, in general, with increasing diameter, the critical buckling strain has a decreasing trend. For infinite length nanotubes, the critical buckling strain of $\left(n,n/2\right)$ nanotubes have higher trend of changes in smaller diameters. In addition, initially the critical buckling strain decreases with higher rate as the diameter increases and at larger diameters the trend of change decreases. At very small diameters the critical buckling strain diameters of $\left(n,n/2\right)$ nanotubes is higher than zigzag and armchair nanotubes, and with increasing diameter the buckling strain of zigzag nanotubes would be larger than armchair nanotubes, and the buckling strain of armchair nanotubes would be larger than $\left(n,n/2\right)$ nanotubes. The amount of the critical buckling strain variations of the $\left(n,n/2\right)$ nanotubes is larger than the zigzag and armchair nanotubes due to the diameter. The ratio of critical buckling strain variations to nanotube diameters is always greater than one, and at diameters lower than 25 angstroms there is an increasing trend and which is reversed at diameters higher than 25 angstroms. As the diameter increases, the critical buckling strain of $\left(n,n/2\right)$ nanotubes obtained by wrapping 2a armchair nanosheets has a decreasing trend, and higher critical buckling strain and diameter-dependent changes compared to infinite length nanotubes. Compared to zigzag and armchair nanotubes obtained by wrapping similar nanosheets and with identical diameters, the critical buckling strain is lower and diameter-dependent changes are higher. The ratio of critical buckling strain to nanotube diameter changes is always greater than one and in the diameters lower than 7 angstroms there is a decreasing trend and in the diameters higher than 7 angstroms, it is reversed. For $\left(n,n/2\right)$ nanotubes obtained by wrapping 3a armchair nanosheets, the critical buckling strain has a decreasing trend with increasing the diameter and has a larger buckling strain and larger diameter-dependent changes compared to similar nanotubes with infinite lengths and compared to similar nanotubes with shorter lengths, they have lower buckling strain and diameter-dependent changes. Compared to zigzag and armchair nanotubes with similar diameters obtained by wrapping similar nanosheets, they have a lower critical buckling strain and higher diameter-dependent changes. The ratio of critical buckling strain to nanotube diameter changes is always greater than one and in the diameters lower than 7 angstroms there is a decreasing trend and in the diameters higher than 7 angstroms, it is reversed. For $\left(n,n/2\right)$ nanotubes obtained by wrapping 5a and 8a armchair nanosheets, the critical buckling strain and diameter-dependent changes have a decreasing trend with increasing diameter and length of the structure, and compared to the infinite length nanotubes, the critical buckling strain and diameter-dependent variations are larger. Compared to similar zigzag and armchair nanotubes, they have lower critical buckling strain and larger diameter-dependent and critical buckling strain variations. By studying $\left(n,n/2\right)$ nanotubes obtained by wrapping 2z zigzag nanosheets, it was found that the critical buckling strain decreasing trend is valid, and compared to infinite length nanotubes, they almost has a similar critical buckling strain and higher diameter-dependent variations. Compared to $\left(n,n/2\right)$ nanotubes obtained by wrapping 2a armchair nanosheets they have lower critical buckling strain and smaller diameter dependent changes. Compared to zigzag and armchair nanotubes obtained by wrapping similar nanosheets, the critical buckling strain is lower and diameter dependent changes are higher. The ratio of critical buckling strain to nanotube diameter changes is always greater than one and in the diameters lower than 7 angstroms there is a decreasing trend and in the diameters higher than 7 angstroms, it is reversed. The critical buckling strain for $\left(n,n/2\right)$ nanotubes obtained by wrapping 3z zigzag nanosheets has a decreasing trend and lower critical buckling strain and larger diameter-dependent variations compared to infinite length nanotubes. Compared to nanotubes with smaller length, they also have lower critical buckling strain and diameter-dependent changes and Compared to nanotubes obtained by wrapping armchair nanosheets with similar length and diameter, they have lower buckling strain and diameter-dependent variations. Compared to zigzag and armchair nanotubes with similar lengths and diameters wrapped in identical plates, they have smaller critical buckling strain and larger diameter dependent changes. Compared to zigzag and armchair nanotubes with similar lengths and diameters obtained by wrapping identical nanosheets, they have smaller critical buckling strain and higher diameter dependent changes. The ratio of critical buckling strain variations to nanotube diameters is always greater than one and in diameters lower than of 23 angstroms there is an increasing trend and when the diameter goes above 23 angstroms it experiences a decreasing trend. For $\left(n,n/2\right)$ nanotubes obtained by wrapping 5z and 9z armchair nanosheets, the critical buckling strain and diameter-dependent changes have a decreasing trend with increasing diameter and an increasing trend with increasing structure length and compared to the infinite nanotubes they have lower critical buckling strain and larger diameter dependent changes. Moreover, compared to zigzag and armchair nanotubes with similar lengths and diameters obtained by wrapping identical nanosheets, they have lower critical buckling strain and larger diameter dependent changes.

**Table 6:** Critical buckling strain results of $\left(n,n/2\right)$ nanotubes with finite and infinite lengths obtained by wrapping armchair and zigzag nanosheets.

	Chirality	(2, 1)	(4, 2)	(6, 3)	(8, 4)	(10, 5)	(14, 7)	(18, 9)	(22, 11)	(26, 13)	(30, 15)
	Diameter	0.2072	0.4144	0.6215	0.8287	1.0359	1.4502	1.8646	2.2789	2.6933	3.1076
	Variation percentage	0	100	200	300	400.01	600.01	800.01	1000	1200	1400
Infinite length	Critical strain	0.3156	0.1251	0.0744	0.051	0.0371	0.0214	0.013	0.0097	0.0105	0.0091
	Variation percentage	0	− 152.35	− 323.91	− 518.9	− 750.57	− 1373	− 2333	− 3147	− 2905	− 3368
	Ratio of strain changes to diameter changes		− 1.5234	− 1.6195	− 1.73	− 1.8764	− 2.287	− 2.916	− 3.146	− 2.421	− 2.405
2a	Critical strain	0.5386	0.1611	0.0961	0.068	0.0518	0.0342	0.0245	0.0183	0.0141	0.0111
	Length to diameter ratio ($L/D$)	35.63229	17.81614	11.87934	8.909147	7.127145	5.091029	3.959568	3.239725	2.741251	2.375792
	Variation percentage	0	− 234.42	− 460.77	− 692	− 939	− 1474	− 2097	− 2837	− 3734	− 4770
	Ratio of strain changes to diameter changes		− 2.3441	− 2.3038	− 2.307	− 2.3475	− 2.456	− 2.621	− 2.837	− 3.111	− 3.407
3a	Critical strain	0.4603	0.1522	0.0922	0.0653	0.0496	0.0323	0.0228	0.0168	0.0129	0.0098
	Length to diameter ratio ($L/D$)	59.38702	29.69351	19.79886	14.84855	11.87855	8.48503	6.599265	5.39953	4.568741	3.959644
	Variation percentage	0	− 202.44	− 399.23	− 605.3	− 828.38	− 1325	− 1918	− 2638	− 3479	− 4621
	Ratio of strain changes to diameter changes		− 2.0243	− 1.9961	− 2.018	− 2.0709	− 2.208	− 2.397	− 2.638	− 2.899	− 3.301
5a	Critical strain	0.377	0.1382	0.0835	0.0586	0.044	0.0276	0.0187	0.0133	0.0092	0.0093
	Length to diameter ratio ($L/D$)	106.8969	53.44843	35.63802	26.72744	21.38144	15.27309	11.8787	9.719176	8.223752	7.127375
	Variation percentage	0	− 172.83	− 351.32	− 543.5	− 756.8	− 1268	− 1917	− 2737	− 4011	− 3954
	Ratio of strain changes to diameter changes		− 1.7282	− 1.7566	− 1.812	− 1.8919	− 2.114	− 2.396	− 2.737	− 3.343	− 2.824
8a	Critical strain	0.3677	0.1382	0.084	0.0585	0.0438	0.0275	0.0184	0.0131	0.0099	0.0092
	Length to diameter ratio ($L/D$)	178.1611	89.08055	59.39659	44.54565	35.63566	25.4551	19.7978	16.1986	13.70623	11.87894
	Variation percentage	0	− 165.96	− 337.64	− 528.5	− 738.83	− 1239	− 1894	− 2713	− 3625	− 3896
	Ratio of strain changes to diameter changes		− 1.6595	− 1.6882	− 1.762	− 1.847	− 2.065	− 2.367	− 2.713	− 3.021	− 2.783
2z	Critical strain	0.3944	0.1239	0.0737	0.051	0.0381	0.0235	0.0156	0.0108	0.0091	0.0093
	Length to diameter ratio ($L/D$)	34.28721	17.14361	11.43091	8.572837	6.858104	4.898848	3.810099	3.117429	2.637772	2.286108
	Variation percentage	0	− 218.36	− 435.2	− 673.3	− 934.6	− 1578	− 2423	− 3548	− 4253	− 4141
	Ratio of strain changes to diameter changes		− 2.1835	− 2.176	− 2.244	− 2.3364	− 2.63	− 3.029	− 3.548	− 3.544	− 2.958
3z	Critical strain	0.3285	0.1155	0.0686	0.0469	0.0343	0.0202	0.0125	0.0091	0.0093	0.0092
	Length to diameter ratio ($L/D$)	54.85951	27.42975	18.28944	13.71653	10.97296	7.838153	6.096155	4.987885	4.220432	3.657771
	Variation percentage	0	− 184.38	− 378.88	− 600.6	− 856.92	− 1530	− 2520	− 3506	− 3436	− 3467
	Ratio of strain changes to diameter changes		− 1.8437	− 1.8943	− 2.002	− 2.1422	− 2.55	− 3.15	− 3.506	− 2.863	− 2.476
5z	Critical strain	0.322	0.1193	0.071	0.0484	0.0354	0.0207	0.0127	0.0091	0.009	0.009
	Length to diameter ratio ($L/D$)	96.00381	48.00191	32.00642	24.00385	19.20262	13.71672	10.66823	8.728768	7.385731	6.401078
	Variation percentage	0	− 169.88	− 353.49	− 564.9	− 810.95	− 1456	− 2444	− 3450	− 3466	− 3474
	Ratio of strain changes to diameter changes		− 1.6987	− 1.7674	− 1.883	− 2.0273	− 2.427	− 3.054	− 3.45	− 2.888	− 2.481
9z	Critical strain	0.3256	0.1236	0.074	0.0505	0.037	0.022	0.0136	0.0098	0.0097	0.0091
	Length to diameter ratio ($L/D$)	178.2932	89.14662	59.44064	44.57869	35.66209	25.47398	19.81249	16.21061	13.71639	11.88775
	Variation percentage	0	− 163.45	− 340.11	− 544.5	− 779.86	− 1381	− 2296	− 3232	− 3260	− 3462
	Ratio of strain changes to diameter changes		− 1.6345	− 1.7005	− 1.815	− 1.9496	− 2.302	− 2.869	− 3.232	− 2.716	− 2.473

The results in Tables 4, 5 and 6, also show that by increasing the length to diameter ratio, for a given chirality that leads to a particular diameter, the critical buckling strain has a decreasing trend. To compare the strength of the structure based on the length to diameter ratio, the diameter of the nanotubes must be equal because the ratio of length to diameters with dissimilar diameters has a different structural physics, that in addition to quantum effects of fine scaling, structure physics also affects the critical buckling of structure and no accurate comparison could be made.

Table 7 presents the critical buckling strain results for nanotubes with finite length and diameter, obtained by wrapping different nanosheets. In this table, in order to compare the quantum effects of finite scaling on the nanotubes with specific length, diameters are chosen in a way that covers the range of 5 to 30 angstroms, and for each specific diameter, the zigzag, armchair and nanotubes with chirality of $\left(n,n/2\right)$ are presented. For nanotubes with a specified length obtained by wrapping armchair nanosheets, the critical buckling strain variations of the two armchair and zigzag structures are closer to each other at smaller diameters, which increase at larger diameters, and at much larger diameters where the structure of nanotubes approaches the nanosheet structure, these critical buckling strain changes decrease. The critical buckling strain variations of armchair and $\left(n,n/2\right)$ nanotubes in smaller diameters, increase as the diameter increases, and have a decreasing trend at larger diameters. As the length of the structure increases, differences between different chiralites become more specific and there is an increasing trend. At larger lengths and diameters, the intensity of change of the critical buckling strain decreases, and this trend confirms that as the diameter increases, the strain would eventually reaches the value of nanosheets, and at larger lengths, it approaches the critical buckling strain of infinite length nanotubes. For nanotubes with specific lengths and diameters obtained by wrapping zigzag nanosheets, the results show that, same as the nanotubes obtained by wrapping armchair nanosheets, the critical buckling strain difference of nanotubes with different chirality increases with increasing length and diameter and at larger lengths and diameters, the intensity of this increasing trend decreases. By comparing the critical buckling strain of nanotubes obtained by wrapping armchair and zigzag nanosheets it was found that the critical buckling strain difference of armchair and zigzag nanotubes, in nanotubes obtained by wrapping zigzag nanosheets was higher than the ones obtained from armchair nanosheets. In addition the critical buckling strain difference of the armchair and $\left(n,n/2\right)$ nanotubes has an increasing trend at smaller diameters and decreases at larger diameters.

**Table 7:** Critical buckling strain of nanotubes with finite length and diameter wrapped obtained by wrapping different nanosheets.

	Chirality	Armchair	Zigzag	Chirality of $(n,n/2)$	Armchair	Zigzag	Chirality of $(n,n/2)$	Armchair	Zigzag	Chirality of $(n,n/2)$	Armchair	Zigzag	Chirality of $(n,n/2)$
	Diameter	0.4069	0.3915	0.4144	0.9494	1.018	1.0359	2.0344	2.0359	2.0717	2.9838	2.9756	2.9004
	Variation percentage	0	− 3.7751	1.8359	0	7.2225	9.109	0	0.0742	1.8354	0	− 0.276	− 2.794
2a	Critical strain	0.181	0.1795	0.1611	0.0698	0.0689	0.0518	0.0303	0.04	0.021	0.0169	0.0313	0.0124
	Variation percentage	0	− 0.8179	− 10.997	0	− 1.275	− 25.73	0	32.036	− 30.81	0	85.765	− 26.51
3a	Critical strain	0.1723	0.1753	0.1522	0.0681	0.0699	0.0496	0.0268	0.0424	0.0197	0.0152	0.0339	0.0113
	Variation percentage	0	1.7468	− 11.682	0	2.6424	− 27.22	0	58.038	− 26.41	0	122.26	− 25.67
5a	Critical strain	0.1602	0.1643	0.1382	0.062	0.0677	0.044	0.0229	0.0415	0.0156	0.0119	0.0335	0.0091
	Variation percentage	0	2.5657	− 13.74	0	9.129	− 29.03	0	81.144	− 31.95	0	181.38	− 23.66
8a	Critical strain	0.162	0.1663	0.1382	0.0622	0.0695	0.0438	0.0227	0.0432	0.0155	0.0115	0.0351	0.0093
	Variation percentage	0	2.6854	− 14.661	0	11.652	− 29.56	0	90.388	− 31.79	0	205.31	− 19.32
2z	Critical strain	0.1401	0.1414	0.1239	0.0533	0.0533	0.0381	0.0193	0.03	0.013	0.0122	0.0229	0.0097
	Variation percentage	0	0.9854	− 11.546	0	− 0.019	− 28.49	0	55.412	− 32.78	0	88.414	− 20.62
3z	Critical strain	0.1337	0.1371	0.1155	0.0498	0.0534	0.0343	0.016	0.0306	0.0097	0.0091	0.0237	0.0092
	Variation percentage	0	2.5125	− 13.617	0	7.3524	− 31.04	0	90.818	− 39.48	0	159.82	0.4391
5z	Critical strain	0.1397	0.1441	0.1193	0.0519	0.0576	0.0354	0.0166	0.034	0.01	0.0091	0.0268	0.0092
	Variation percentage	0	3.1861	− 14.57	0	10.903	− 31.9	0	104.45	− 40.08	0	195.48	1.1025
9z	Critical strain	0.1458	0.1502	0.1236	0.054	0.0615	0.037	0.0175	0.0372	0.0109	0.0091	0.0297	0.0094
	Variation percentage	0	2.9975	− 15.241	0	13.807	− 31.52	0	112.03	− 37.69	0	225.11	3.0702

## Validation with other works

To show the validity of the current study, in addition to Table 3 and Fig. 13, we also represent some of the available data from other literatures in Tables 8, 9, 10 and 11.

**Table 8:** Comparison between exact and approximate buckling loads ${P}_{cr}$ (nN) for the simply supported based on nonlocal Euler–Bernoulli beam model.

$e_{0}a$ (nm)	0			1			2		
$L/d$	${P}_{cr}$ (exact)^107^	${P}_{cr}$ (DTM)^60^	${P}_{cr}$^108^	${P}_{cr}$ (exact)^107^	${P}_{cr}$ (DTM)^60^	${P}_{cr}$^108^	${P}_{cr}$ (exact) ^107^	${P}_{cr}$ (DTM)^60^	${P}_{cr}$^108^
10	4.8447	4.8447	4.8447	4.4095	4.4095	4.4095	4.0460	4.0460	4.0460
12	3.3644	3.3644	3.3644	3.1486	3.1486	3.1486	2.9588	2.9588	2.9588
14	2.4718	2.4718	2.4718	2.3533	2.3533	2.3533	2.2456	2.2456	2.2456
16	1.8925	1.8925	1.8925	1.8222	1.8222	1.8222	1.7569	1.7569	1.7569
18	1.4953	1.4953	1.4953	1.4511	1.4511	1.4511	1.4094	1.4094	1.4094
20	1.2112	1.2112	1.2112	1.1821	1.1821	1.1821	1.1542	1.1542	1.1542

$e_{0}a$ shows the effects of nonlocal parameter, *L* is the length and *d* is rod diameter.

**Table 9:** Comparison of axial buckling load of the CNT embedded in Winkler, Pasternak and Kerr’s medium.

	$e_{0}a$ = 0 nm		$e_{0}a$ = 1 nm		$e_{0}a$ = 2 nm	
	$L/d$ = 5	$L/d$ = 10	$L/d$ = 5	$L/d$ = 10	$L/d$ = 5	$L/d$ = 10
Without medium^107^	19.3789	4.8447	13.8939	4.4095	7.5137	3.4735
Without medium^108^	19.3789	4.8447	13.8939	4.4095	7.5137	3.4735
Winkler medium^108^	39.2733	9.8183	33.7882	9.3831	27.4081	8.44710
Pasternak^108^	58.9082	14.7271	53.4232	14.2919	47.0430	13.3558
Kerr medium^108^	45.3062	11.3265	39.8211	10.8913	33.4410	9.95530

$e_{0}a$ shows the effects of nonlocal parameter, *L* is the length and *d* is rod diameter.

**Table 10:** MD buckling results for SWCNTs under axial load^109^.

SWCNT (5, 5)			SWCNT (10, 10)			SWCNT (15, 15)			SWCNT (20, 20)		
D = 0.678 nm			D = 1.356 nm			D = 2.034 nm			D = 2.713 nm		
$L/d$	${P}_{cr}$ (nN)	${\varepsilon }_{cr}$	$L/d$	${P}_{cr}$ (nN)	${\varepsilon }_{cr}$	$L/d$	${P}_{cr}$ (nN)	${\varepsilon }_{cr}$	$L/d$	${P}_{cr}$ (nN)	${\varepsilon }_{cr}$
2.0	76.9	0.0734	1.0	86.8	0.0482	1.0	83.3	0.0336	1.5	79.2	0.0255
3.1	65.1	0.0643	1.5	79.8	0.0450	1.4	77.7	0.0316	1.8	78.1	0.0249
4.2	61.5	0.0614	2.1	79.5	0.0443	1.6	77.6	0.0315	2.0	78.1	0.0249
4.9	59.1	0.0595	2.4	77.2	0.0432	2.0	77.6	0.0314	2.2	78.1	0.0248
6.0	57.1	0.0579	3.0	76.9	0.0430	2.4	76.7	0.0310	2.6	77.9	0.0248
7.0	55.6	0.0567	3.5	74.8	0.0421	2.7	75.7	0.0306	3.0	77.9	0.0248
8.1	55.1	0.0562	4.1	73.5	0.0402	3.0	75.7	0.0306	3.5	77.8	0.0248
8.9	48.9	0.0520	4.4	70.0	0.0400	3.3	74.5	0.0303	3.8	77.7	0.0247
9.9	41.1	0.0454	5.0	67.8	0.0389	4.0	74.5	0.0302	4.0	76.9	0.0245
			6.1	65.2	0.0376	4.6	74.5	0.0302	4.5	76.2	0.0242
			7.0	62.9	0.0366	5.0	73.9	0.0300			
			7.5	62.1	0.0363	5.4	73.5	0.0298			
			8.0	61.6	0.0359	6.0	73.0	0.0297			
			9.0	60.6	0.0355	6.7	70.6	0.0288			
			10.0	59.8	0.0351						
			20.0	18.6	0.0131

**Table 11:** MD buckling results for SWCNTs with $L/d$ ≤ 10 under torsion^109^.

SWCNT (5, 5)			SWCNT (10, 10)			SWCNT (15, 15)			SWCNT (20, 20)		
D = 0.678 nm			D = 1.356 nm			D = 2.034 nm			D = 2.713 nm		
$L/d$	${{\text{T}}}_{cr}$ (nN-nm)	${\uptheta }_{cr}$ (rad)	$L/d$	${{\text{T}}}_{cr}$ (nN-nm)	Θcr (rad)	$L/d$	${{\text{T}}}_{cr}$ (nN-nm)	${\uptheta }_{cr}$ (rad)	$L/d$	${{\text{T}}}_{cr}$ (nN-nm)	${\uptheta }_{cr}$ (rad)
2.0	16.0	0.5760	1.0	43.6	0.2007	1.0	53.3	0.1222	1.5	46.0	0.0960
3.1	11.2	0.6371	1.5	30.6	0.2269	1.4	43.8	0.1396	1.8	42.0	0.1047
4.2	8.9	0.6894	2.1	24.6	0.2531	1.6	39.1	0.1484	2.0	39.2	0.1135
4.9	8.0	0.7330	2.4	22.9	0.2793	2.0	35.3	0.1658	2.2	38.7	0.1222
6.0	7.2	0.8116	3.0	20.2	0.3054	2.4	31.0	0.1745	2.6	36.8	0.1309
7.0	6.7	0.8988	3.5	19.5	0.3491	2.7	27.8	0.1833	3.0	31.7	0.1396
8.1	6.5	0.9948	4.1	18.5	0.3841	3.0	26.7	0.1920	3.5	29.0	0.1484
8.9	6.3	1.0646	4.4	16.8	0.3843	3.3	24.6	0.2007	3.8	28.4	0.1571
9.9	6.2	1.1694	5.0	14.8	0.3844	4.0	22.6	0.2270	4.0	27.7	0.1658
			6.1	12.5	0.4014	4.6	21.8	0.2531	4.5	26.3	0.1745
			7.0	11.2	0.4189	5.0	20.9	0.2618			
			7.5	10.8	0.4363	5.4	20.8	0.2793			
			8.0	10.4	0.4538	6.0	19.0	0.2880			
			9.0	9.9	0.4800	6.7	16.8	0.2881			
			10.0	9.6	0.5236						
			20.0	8.4	0.9250						

It should be noticed that since the effect of fine scaling has never been studied before, the available data is used to validate the none-fine scale results, which shows a good agreement. In different articles, the results show that the critical buckling force decreases with increasing the ratio of length to diameter, and the critical buckling force, which is directly related to the critical buckling strain, decreases with increasing the ratio of length to diameter. In addition, with increasing the ratio of length to diameter, the buckling strength of the structure decreases. The results and the process of its changes in this article are in good agreement with other literatures. It should be noted that for a nanotube with different lengths and diameters, the length to diameter ratio could be the same, which allows the buckling strain to be different, and for a more accurate comparison, the length to diameter ratio for structures with the same diameter should be compared to each other. Tables 8 and 9 show the comparison between exact and approximate buckling loads ${P}_{cr}$ (nN) for the simply supported based on nonlocal Euler–Bernoulli beam model and the comparison of axial buckling load of the CNT embedded in Winkler, Pasternak and Kerr’s medium, respectively. In addition, $e_{0}a$ shows the effects of nonlocal parameter, *L* is the length and *d* is rod diameter. Tables 10 and 11 show the MD buckling results for SWCNTs under axial load and MD buckling results for SWCNTs with L/D ≤ 10 under torsion, respectively. In these two tables, ${\varepsilon }_{cr}$ is the critical buckling strain,${P}_{cr}$ is the critical buckling load and ${{\text{T}}}_{cr}$ and ${\uptheta }_{cr}$ are the Critical buckling torque and critical end rotation, respectively.

## Conclusion

In this paper, the buckling behavior of carbon nanotubes with different chirality and lengths under axial loading were investigated for the first time using the quantum mechanics and molecular mechanics methods. By comparing the buckling behavior of nanotubes at different lengths and diameters, the effect of quantum effects on the buckling behavior of one-dimensional (infinite length nanotubes) and zero-dimensional (finite length nanotubes) nanostructures can be investigated. First, by combining quantum mechanics and molecular mechanics, molecular mechanics coefficients were obtained for finite and infinite length nanostructures, and then, by using molecular mechanics, the buckling behavior of finite and infinite nanotubes was investigated with respect to their diameters. In addition, besides studying the quantum effects of fine scaling dependence of nanostructures on the longitudinal changes, the quantum effects of atomic arrangement have also been investigated on the buckling behavior of the nanostructures in such a way that fine-scale nanostructures with identical lengths obtained from nanosheets with different atomic arrangement have also been studied. The results show that the critical buckling strain of the CNTs, which reflects the buckling behavior of the nanotubes, is influenced by the atomic arrangement and the type of structure, which are wrapped to make nanotubes, as well as the length of the structure. The results show that, in general, nanotubes with zigzag atomic arrangement are more resistant to axial load, which leads to buckling strain, compared to nanotubes with different chiralities and with changes in diameter and length, they show less variation. With increasing length for nanotubes obtained by wrapping armchair nanosheets, the buckling strain changes are more affected by the quantum effects than the nanostructures obtained by wrapping zigzag nanosheets. In other words, it is shown that the buckling behavior of structure with zigzag atomic arrangement is less affected by the quantum effect than the structure with armchair atomic arrangement. For nanotubes obtained by wrapping armchair nanosheets, the critical buckling strain decreases with increasing the length of the nanotubes. In other words, the results show that the smaller nanotubes can withstand higher loadings before entering the buckling process. As a conclusion, it should be noted that the buckling behavior of nanostructures at very small dimensions is highly dependent on the length, and atomic arrangement of the structure. In addition, at a very small scale, the quantum effects have an important impact on the behavior and properties of the nanostructures that if not considered in the calculations done by scientists, it would greatly cause errors in their conclusions and failure to achieve their intended purpose.

## Data Availability

The datasets generated and/or analysed during the current study are not publicly available due technical or time limitations but are available from the corresponding author on reasonable request.
